# RAD51 stabilizes neutrophil extracellular traps to compartmentalize inflammation

**DOI:** 10.1126/science.aed9286

**Published:** 2026-08-20

**Authors:** Lorenza Iolanda Tsansizi, Sophie Yihan Guan, Iker Valle Aramburu, Rajvee Shah Punatar, Thomas J. Williams, Yihe E. Qiao, Anna Reed, Darius Armstrong-James, Stephen C. West, Venizelos Papayannopoulos

**Affiliations:** 1 Antimicrobial Defence lab, The Francis Crick Institute, London, UK; 2 DNA Recombination and Repair lab, The Francis Crick Institute, London, UK; 3 Department of Infectious Disease, Faculty of Medicine, Imperial College, London, United Kingdom; 4 Respiratory & Transplant Medicine, Royal Brompton and Harefield Hospitals, Part of Guy’s and St Thomas’ NHS Foundation Trust and Imperial College London, London, UK; 5 Department of Respiratory Medicine, Royal Brompton and Harefield Hospitals, Guy’s and St. Thomas’ NHS Foundation Trust, London, United Kingdom

## Abstract

Neutrophil extracellular traps (NETs) feature a branched chromatin architecture whose origin and function remain unknown. We found that NET branching is mediated by RAD51, a protein generating DNA junctions during DNA recombination repair. Pharmacological inhibition, RAD51 knock-down or GEN1 and RuvC resolvase treatment reduced branching and destabilized NETs, while RAD51 upregulation by different stimuli generated NETs with variable stability. RAD51 inhibition during murine pulmonary *Aspergillus fumigatus* infection dismantled NETs and reduced lung cytokines. However, the increased accumulation of NET components in the circulation, led to IL-6 induction in circulating monocytes that exacerbated type-2 inflammation and asthma. Extracellular plasma DNA correlated with IL-6 and eotaxin in human aspergillosis. By structurally stabilizing NETs, RAD51 compartmentalizes inflammation to thwart aberrant systemic immune activation, linking DNA repair to inflammation.

## Introduction

Inflammation can be driven by a variety of microbial pathogen-associated molecular patterns (PAMPs) and endogenous danger associated molecular patterns (DAMPs). Containment of inflammation to sites of infection is critical to reduce immune pathology. Immune cells control microbial dissemination but the mechanisms that spatially contain DAMPs remain poorly understood.

Neutrophil extracellular traps (NETs) are large web-like structures composed of decondensed chromatin and antimicrobial proteins (1–5). NETs are released by neutrophils to neutralize pathogens but are also implicated in diverse physiological and pathological processes (6, 7). NETs trap and control large microbes such as fungal hyphae and parasites extracellularly, requiring NET chromatin to be highly decondensed and to occupy a large volume (3–5). This massive chromatin expansion is achieved via the action of proteases such as neutrophil elastase (NE), cationic proteins such as myeloperoxidase (MPO) and post-translational modifications such as histone citrullination (8–12). How this large mass of extracellular chromatin is stabilized and held together is unclear.

In addition to controlling microbes, NET chromatin is pro-inflammatory through the ability of its histones to activate TLR4 (13, 14). In monocytes, TLR4 histones and DNA act synergistically to induce cytokines (14). Chromatin derived from NETs or other cellular sources promotes inflammation in infected tissues during acute pulmonary fungal infection and the circulation during sepsis or atherosclerosis (14, 15). Moreover, NETs exacerbate allergic asthma in response to viral infection by amplifying type-2 immunity via unknown mechanisms (16). Exacerbated type-2 immunity mediates immune hypersensitisation and pathology associated with chronic exposure to fungal pathogens such as *Aspergillus fumigatus* afflicting millions of patients world-wide (17, 18). Although *A. fumigatus* hyphae are potent inducers of NETosis, the role of NETs in the development of aspergillosis pathology remains poorly understood (3).

The timely degradation of NETs is thought to be important to control inflammation, tissue damage, and disease pathology (19, 20). Plasma DNases dismantle NETs but also disarm nucleosomes by removing DNA and thereby preventing the activation of monocytes (14, 15, 19, 21). NET clearance deficiency has been implicated in a range of disorders, including autoimmune diseases, cardiovascular conditions, and severe infections such as microbial sepsis and COVID-19 pneumonia (19, 20, 22–24).

It is unclear what factors determine the structural integrity of NETs and how that influences their biological function and role in diseases. NET chromatin bears a distinctive structural architecture that distinguishes it from chromatin derived from other cellular sources. In addition to being large, NET chromatin fibres are extensively intertwined (1, 8). The function of this branched chromatin architecture is unknown, but it could serve to stabilize the large extracellular conformation.

During NETosis, neutrophils generate large quantities of reactive oxygen species (ROS) that are required for NET formation by activating a myeloperoxidase-containing complex that mediates the activation and release of NE from granules (25, 26). Furthermore, ROS promote DNA damage leading to the activation of DNA repair pathways (27). The functional significance of DNA repair in NETosis remains poorly understood. RAD51 is a RecA-like ATPase involved in homologous DNA recombination during double strand break repair. RAD51 promotes strand invasion into homologous duplex DNA generating Holiday junction intermediates (28–31). Although extensively studied in cancer, whether RAD51 and its paralogs play a role in immune regulation remains unclear (28, 32). Here, we set out to explore the role of RAD51 in chromatin organization during NET formation and the role of DNA repair in NET biology and the spatial control of inflammation.

## Results

### RAD51 promotes NET chromatin branching and stability

**Figure 1:**
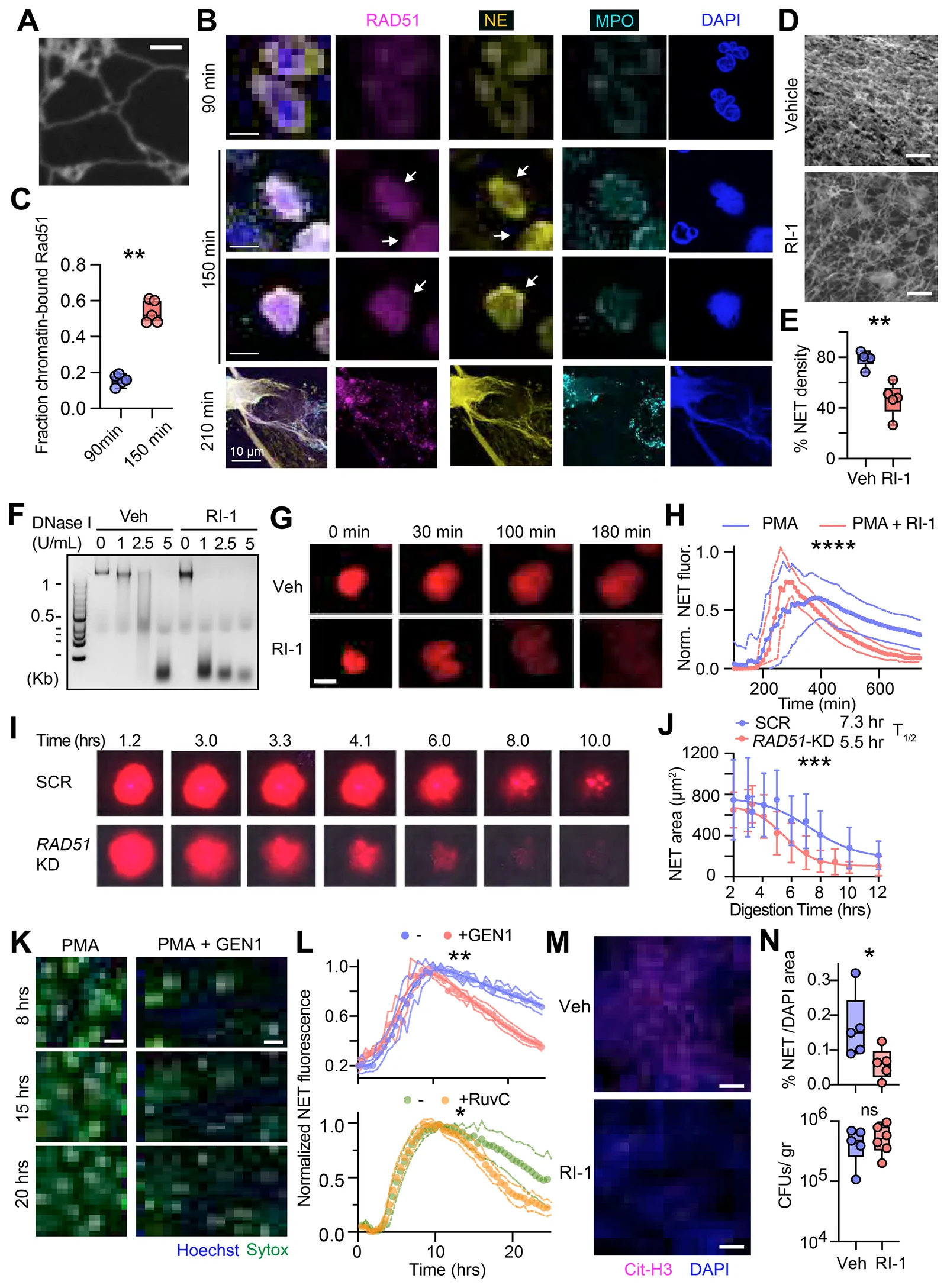
RAD51 cross-links NET chromatin to maintain NET stability (**A**) Transmission electron microscopy of NETs formed by human neutrophils isolated from the blood and stimulated with PMA for 4 hrs. (**B**) Single z-plane micrographs obtained by confocal fluorescence microscopy of human neutrophils activated with PMA at different stages of NETosis, stained for RAD51 (magenta), NE (yellow), MPO (cyan), and DNA (DAPI, blue). Arrows depict RAD51 translocated to the nucleus alongside NE. (**C**) Quantification of the fraction of RAD51 colocalizing with chromatin (DAPI) in individual human blood neutrophils at 90 min (N=6) and 150 min (N=5) post-PMA stimulation. (**D**) Electron microscopy of NET chromatin formed by human neutrophils stimulated with PMA in the presence of vehicle (DMSO) or RI-1. (**E**) Quantification of NET density in multiple electron micrographs as shown in (D). Each point represents the mean NET density an individual images (N=5). (**F**) Agarose electrophoresis of NETs formed in the presence of vehicle or RI-1 and treated with increasing concentrations of DNase I. (**G**) Timelapse microscopy of degradation of NETs formed by human blood neutrophils in the presence of 3% human plasma treated with vehicle (DMSO) or RI-1. NETs were stained with Sytox Green and tracked every 5 min for 180min post-NET formation. Scale bar: 50μm. (**H**) Changes in NET fluorescence intensity over time used to calculate NET dissociation curves in the presence or absence of RI-1. Each graph is the aggregate of approximately N=400 NETs per sample tracked individually with standard deviation. Representative of 5 individual experiments. (**I**) Timelapse microscopy of NET decay over 10 hrs released by HoxB8-derived murine neutrophils expressing either scrambled (SCR) or *RAD51* knock-down RNA, in the presence of DNase I. NETs were stained with Sytox Green. (**J**) Changes in NET area over time in (I). The mean NET half-life SCR (7.3 hrs) and RAD51-KD (5.5.hr) was obtained by fitting non-linear regression curves to the raw data. Scr N=20 and *RAD51-KD* N=36. Representative of 2 biological repeats and 2 independent experiments. (**K**) Representative timelapse microscopy images from human neutrophils activated with PMA and treated with either vehicle or GEN1, monitored every 30 min for 24 hrs, depicting NETs that have formed after 8, 14, 20 hrs of PMA stimulation. Nuclei were stained with Hoechst (blue) and Sytox Green (green). (**L**) Quantification of changes in NET chromatin density in the presence of vehicle, GEN1 or RuvC monitored by measuring NET Sytox signal intensity by microscopy of N=40-100 individual NETing events across 4 different timelapse movies per sample. Data fitted by non-linear regression. (**M**) Confocal fluorescence micrographs of lungs of WT mice treated with either vehicle or RI-1 and infected with *wt A. fumigatus* 24 hrs post-infection, stained for citrullinated histone H3 (Cit-H3, magenta), and DNA (DAPI, blue). (**N**) (upper panel) Quantification of total NET area normalized to total lung tissue area (DAPI) per image in 4 images per mouse for N=5 control and N=6 RI-1 treated mice (M). (lower panel) Fungal load (CFU) for the corresponding animals. Each data point represents an individual mouse. Scale bars: (A) 20 nm, (B) 10 μm, (D) 0.5 μm, (K) 30 μm, (M) 50 μm. Representative of (B-F, I-N) 2 and (G, H) 6 individual experiments. Statistics by one-way Anova (C, E, N), or by two-way Anova (H, J, L), ****p<0.0001, ***p<0.001, **p<0.01, *p<0.05, ns>0.05.

To investigate whether NET chromatin branching implicated DNA recombination repair, we examined in detail the architecture of NET chromatin by scanning electron microscopy. NETs released by human blood neutrophils in response to phorbol myristate acetate (PMA), a potent NET-inducing signal, contained a dense array of interlinked DNA strands that when imaged by electron microscopy, resembled the denatured forms of four-way DNA recombination intermediates called Holliday junctions (HJ) (Fig. 1A) (33, 34). To explore whether RAD51 was implicated in NET architecture, we examined whether RAD51 was sequestered to the nucleus during PMA-induced NET formation in human primary neutrophils by confocal immunofluorescence microscopy. NE translocates to the nucleus early during NETosis, driving chromatin decondensation prior to cellular rupture, whereas MPO binds to chromatin at late stages of the process (8). RAD51 localized outside the nucleus in resting neutrophils but was sequestered to foci inside the nucleus of cells undergoing NET formation, 150 min post-PMA stimulation (Fig. 1B and 1C). RAD51 translocation occurred concomitantly with the nuclear translocation of NE and remained associated with chromatin fibres after NET release. We also observed colocalization of RAD51 with NETs in the lungs of mice following a pulmonary fungal challenge (Fig. S1B). RAD51 binds to ssDNA which arises when a double strand break is resected to produce single strand tails. Consistently, positive TUNEL staining indicated that NET formation promoted double stranded breaks both in NETs produced by human blood neutrophils *in vitro* and *in vivo* during murine pulmonary fungal infection (Fig. S2A and S2B).

To examine whether RAD51 played a role in NET formation, we employed RI-1, an inhibitor that binds to a critical pocket and is stabilized by a disulfide bond (Fig. S3A) (35). We confirmed that RI-1 inhibited RAD51 using a RAD51-mediated strand exchange assay (Fig. S3B). RI-1 treatment did not interfere with PMA-induced NET formation by human blood neutrophils (Fig. S3C). Instead, electron microscopy revealed that RAD51 inhibition altered the architecture of NETs, reducing the frequency of branching and the density of NETs (Fig. 1D and 1E). Blocking RAD51 activity resulted in more exposed NET DNA as indicated increased sensitivity to DNase I endonuclease digestion (Fig. 1F). These findings suggested that RAD51 promoted NET chromatin branching and resistance to endonuclease digestion.

To evaluate whether chromatin branching influenced the structural stability of NETs, we performed time-lapse video microscopy of human neutrophils undergoing PMA-induced NETosis in the presence or absence of DNAse I and monitored the rate of NET degradation. Pharmacological inhibition of RAD51 using RI-1 accelerated the degradation of NETs in the presence of 3% human plasma that contained DNase I (Fig. 1G and 1H). (36). The concentration of RI-1 required for 50% increase in NET destabilization was approximately 30 μM (Fig. S3D). The effects of RAD51 inhibition on NET destabilization depended on DNase I activity, as no differences in NET stability were observed in similar experiments performed in the absence of plasma or DNase I (Fig. S3E). We observed comparable NET destabilisation with B02 that also inhibits RAD51, whereas Rucaparib, an inhibitor of PARP proteins involved in base-excision repair of single stranded breaks had a minimal impact on NET stability (Fig. S3F). Therefore, RAD51 branching increased NET stability, likely requiring more cuts to dismantle the DNA scaffold.

In addition to testing RAD51 inhibitors, we sought to obtain genetic evidence for the role of RAD51 in NET stability. We attempted to genetically ablate RAD51 by using CRISPR in conditionally immortalized HoxB8 hematopoietic stem cells that can be differentiated to neutrophils (37). This approach generated RAD51-deficient progenitors that exhibited poor viability and proliferation, which is consistent with RAD51 being essential for cell survival (Fig. S3G and S3H). To overcome this issue, we employed an inducible shRNA knockdown strategy in murine HoxB8 stem cell-derived neutrophils. We tested 5 shRNA candidates and 2 were effective in suppressing *RAD51* expression (Fig. S3I). To obtain viable terminally differentiated neutrophils, it was necessary to optimise the timing of shRNA induction by aiming at the late stages of the differentiation process. Unlike the human NETs that expanded and lost Sytox-labelled DNA fluorescence uniformly as they disintegrated, the core Sytox-labelled DNA area of HoxB8-derived NETs shrunk and shrivelled as they dissociated. This suggested a non-uniform pattern of dissociation from the periphery towards the centre. Hence, we opted to measure the decrease in NET area instead of the loss in DNA fluorescence intensity. As with RI-1 and other RAD51 inhibitors, NETs formed by HoxB8-derived *RAD51* knockdown neutrophils were of similar size but dissolved more rapidly when incubated in the presence of DNase I compared to control cells receiving a scrambled shRNA control (NET half-life for scrambled control: 7.3 hrs vs *RAD51-KD*: 5.5 hrs) (Fig. 1I and 1J). These findings suggested that the formation of a portion of NET DNA branches required RAD51.

We attempted to detect RAD51-mediated DNA joint molecules in NETs biochemically and evaluate their impact on NET stability, by testing whether NETs were sensitive to extracellular treatment with the structure-selective endonucleases GEN1 and RuvC. These enzymes recognize a range of branched and joint DNA molecules, with GEN1 targeting any non-linear DNA and RuvC having more specificity towards Holliday junctions and D-loops. Time-lapse microscopy analysis indicated that GEN1 or RuvC treatment during NET formation destabilized NETs and accelerated their disassembly (Fig. 1K and 1L). GEN1 was sufficient to destabilize NETs in the absence of plasma endonucleases, whereas the effects of RuvC were prominent in the presence of plasma, as observed with RI-1 or RAD51 KD inhibition strategies that also required plasma. The enzymes did not affect the nuclear DNA from bystander necrotic neutrophils that died without making NETs and remained condensed in the same reaction (Fig. 1K). The difference between the two enzymes suggested that a proportion of NET DNA branches were formed by RAD51-independent mechanisms, and this is consistent with the EM analysis that shows that fewer but still considerable number of DNA branches remain in NETs formed in the presence of RI-1 (Fig. 1D).

We also examined the impact of RAD51 on NET stability *in vivo* using several pulmonary fungal infection models. NET formation during pulmonary *A. fumigatus* or *C. albicans* infection peaks 24 hrs post-infection (3). RI-I treatment dissolved NETs more rapidly in pulmonary infection with wt *A. fumigatus* with most NETs disappearing 24 hrs post-infection despite the comparable fungal burden (Fig. 1M and 1N). Treatment with RI-1 also enhanced NET clearance in WT mice infected intra-tracheally with wt *Candida albicans* (Fig. S4A and S4B) or *Dectin-1* knockout animals infected with yeast-locked *Δhgc1 C. albicans* that promotes NET release due to defective phagocytosis of yeasts (Fig. S4C and S4D) (3). We concluded that RAD51 promoted chromatin interconnections that stabilized the structural integrity of NETs and rendered them more resistant to degradation by plasma endonucleases *in vitro* and *in vivo*.

### Stimulus-dependent induction of RAD51 generates NETs with variable stability

**Figure 2:**
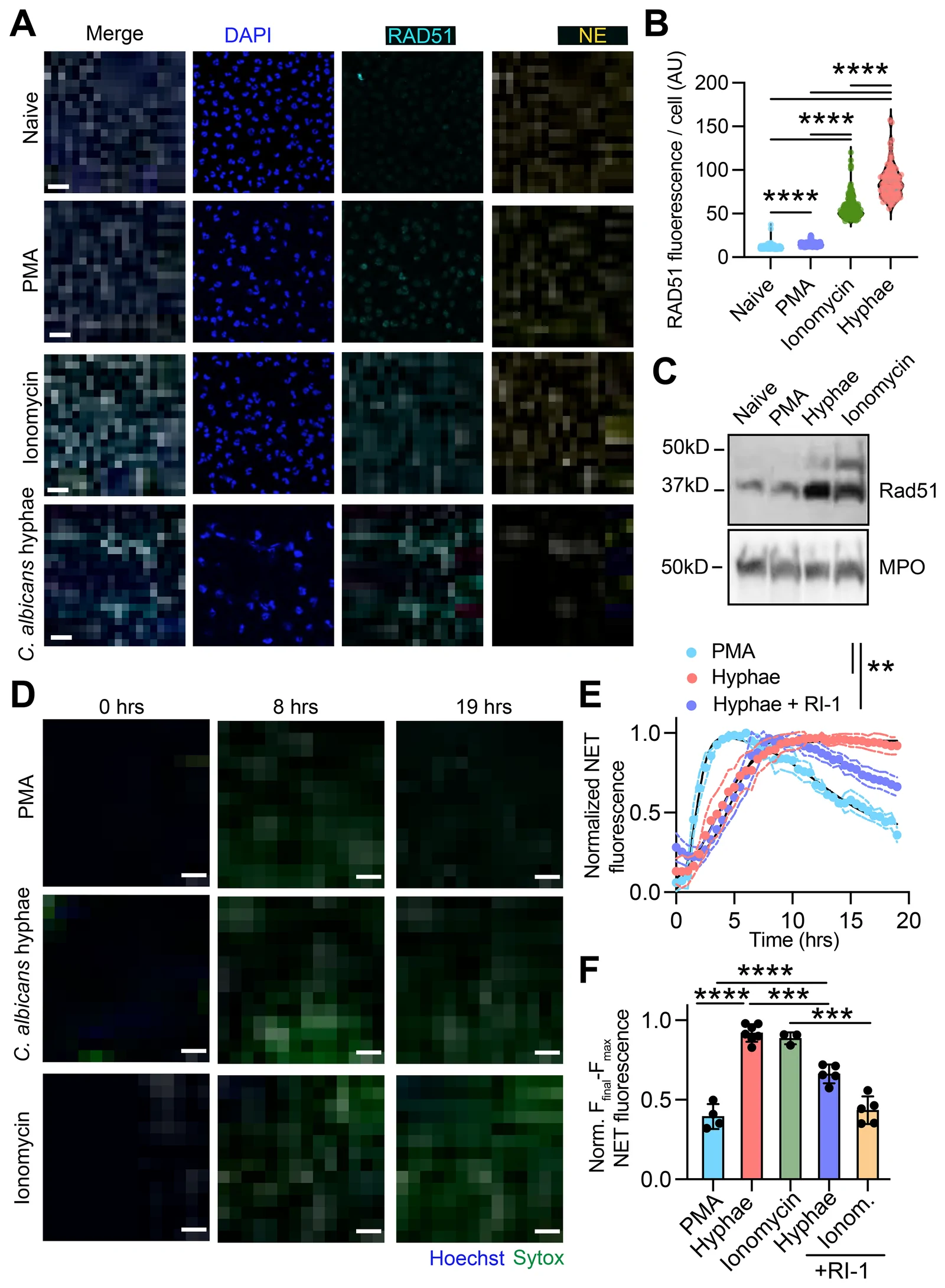
RAD51 expression is differentially upregulated in response to different NET-inducing stimuli. (**A**) Confocal fluorescence micrographs of human blood neutrophils either naïve or stimulated with either heat-inactivated *C. albicans* hyphae, ionomycin or PMA, imaged 90 min post-stimulation. Scale bars: 30 μm. Representative of 2 individual experiments. (**B**) Quantification of RAD51 fluorescence intensity per neutrophil in (A). Individual neutrophils were quantified from multiple micrographs (naïve N=515, PMA N=315, hyphae N=83, ionomycin N=172). (**C**) Western immunoblotting of neutrophil extracts taken after 90min from either naïve cells or stimulated with ionomycin or hyphae, stained for RAD51 and MPO proteins. (**D**) Representative still micrographs of NET formation and degradation by timelapse microscopy of human neutrophils activated with either PMA, *C. albicans* hyphae or ionomycin alone pre-treated with vehicle or RI-1 in the presence of human plasma that contains DNase I. Images were captured every 30 min. Scale bars: 50 μm. (**E**) Quantification of NET degradation in all samples in (E) by quantifying the mean loss of Sytox fluorescence per NET at 24 hrs post stimulation measured by microscopy. Each point is the mean value of approximately 50 NETs in an individual timelapse movie, with PMA N=4, hyphae N=7, Ionomycin N=3, hyphae+RI1 N=5 and ionomycin+RI-1 N=5 movies per sample (technical replicates). Statistics by one-way Anova (B and F), or two-way Anova (E), ****p<0.0001, ***p<0.001, **p<0.01, *p<0.05, ns>0.05.

To evaluate whether RAD51-mediated stability was a general feature of NETs we monitored RAD51 expression in response to other NET-inducing stimuli. PMA, *C. albicans* hyphae and ionomycin upregulated RAD51 expression to variable degrees. Compared to PMA, hyphae and ionomycin induced higher RAD51 protein expression that accumulated in neutrophil nuclei as assessed by immunofluorescence microscopy and western immunoblotting (Fig. 2A-C). To examine whether the differential upregulation of RAD51 in response to different signals impacted NET formation and stability, we measured the rates of NETosis and decay in response to PMA, *C. albicans* hyphae or ionomycin. Notably, NETs induced by hyphae or ionomycin were more stable against DNase I-mediated degradation than NETs formed by PMA stimulation (Fig. 2D-F). Pre-treatment with RI-1 reduced the stability of NETs induced by hyphae or ionomycin. Hence, different stimuli yielded NETs with variable stability by modulating the expression of RAD51. PMA-derived NETs were the most sensitive to degradation, whereas NETs induced by fungi or ionomycin were more resistant and this phenomenon was linked to the higher induction of RAD51.

### RAD51-mediated NET stability spatially controls inflammation

**Figure 3:**
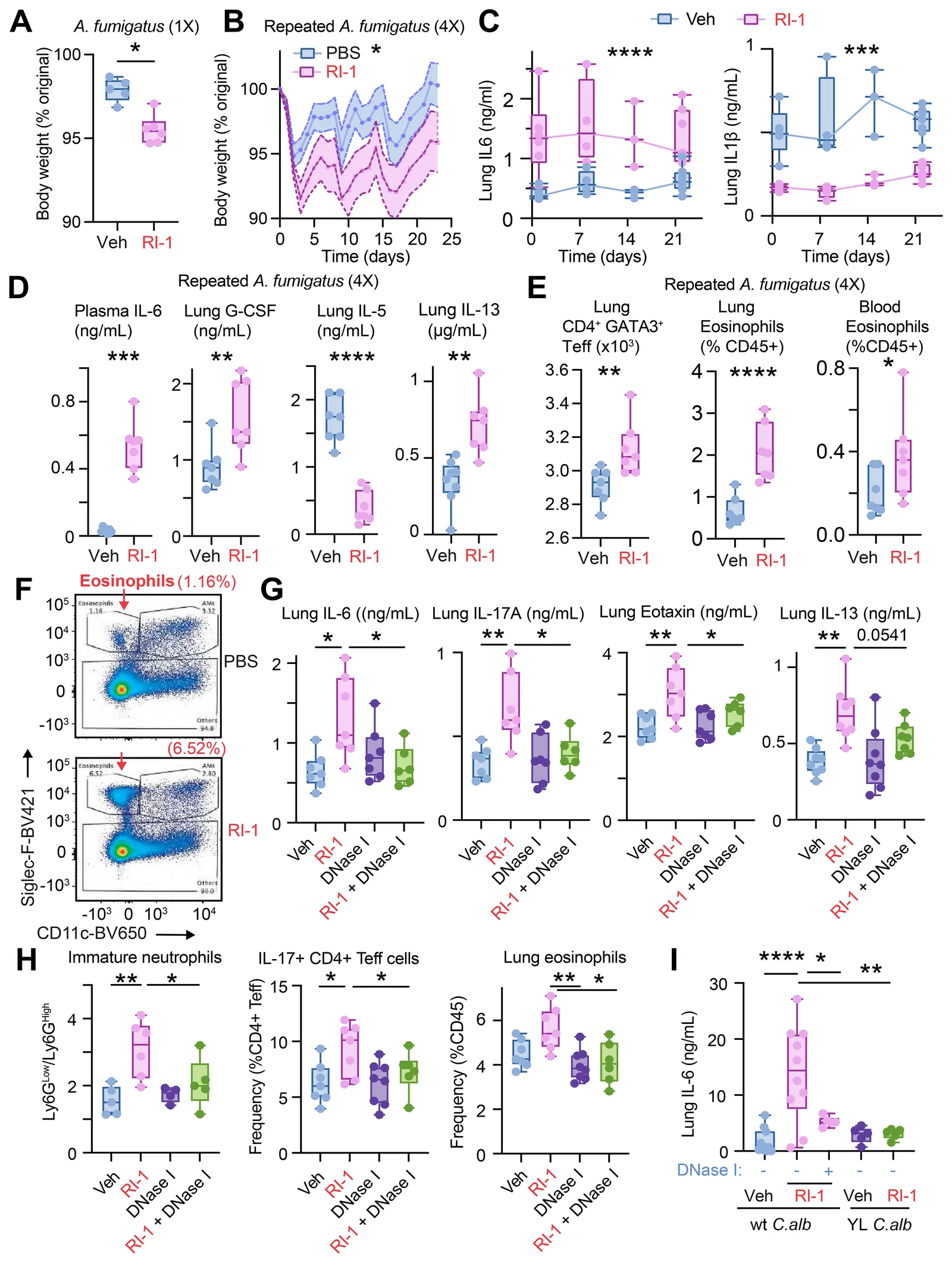
Loss of NET stability alters local and systemic inflammation inducing eosinophilia (**A**) Body weight 24 hrs post-infection, normalized to initial weight of mice treated with either vehicle or RI-1 and infected with a single high dose of *A. fumigatus* (acute model). (**B** to **F**) Mice treated with vehicle or RI-1 and challenged with 4 low doses of *A. fumigatus* (chronic exposure model). Readouts were assessed 24 hrs after the 4rth infection: (**B**) Change in normalized body weight over time. (**C**) Pulmonary concentrations of IL-1β and IL-6 protein. (**D**) Plasma and lung IL-6, IL-5, and IL-13 protein concentrations. (**E**) Lung and blood frequencies of GATA3^+^ Th2 cells and eosinophils. (**F**) Representative flow cytometry plots of eosinophils. Cells were subsequently also gated for CD64 and CD125 to exclude minor non-eosinophil macrophage populations in the counts (gating shown in Fig. S7A). (**G**) Lung and plasma cytokines 24 hrs after infection with a single high dose of *A. fumigatus* and treatment with vehicle, RI-1, DNase I or a combination of RI-1 and DNAse I. (**H**) Ratio of Ly6G^low^ immature over Ly6G^high^ mature neutrophils, IL-17 producing CD4 effector T cells and eosinophils in (G). (**I**) Lung IL-6 concentrations in mice infected with either WT or mutant yeast-locked *Δhgc1 C. albicans*, treated with either vehicle or RI-1 and DNase I. Each data point represents an individual mouse in a single experiment, bars show the mean and 10%-90% percentile and error bars ± SEM. Representative of 3 (A to E) and 2 (G to I) independent experiments. Statistics by one-way or two-way (Fig. 3B) Anova, ****p<0.0001, ***p<0.001, **p<0.01, *p<0.05, ns>0.05.

Given that NET degradation by exogenous DNase I treatment counters inflammatory pathology, we investigated whether RI-1 treatment would yield similar benefits during infection by reducing inflammation. However, mice treated with RI-1 lost more weight 24 hrs after being infected intratracheally with *Aspergillus fumigatus* (Fig. 3A). Similarly, chronic exposure to 4 consecutive challenges with *A. fumigatus* over a period of 4 weeks led to increased weight loss in RI-1 treated animals (Fig. 3B). To understand the mechanism of pathology we investigated the impact of RI-1 on inflammation and immune polarization. RI-1 treatment reduced the concentration of IL-1β but increased the levels of IL-6 in homogenized lung tissues 24 hrs post-infection of both acute and chronic aspergillosis models (Fig. 3C). These changes were also reflected in the plasma cytokine concentrations in the circulation (Fig. 3D). RAD51 inhibition also affected T cell-derived type-2 cytokines, leading to a 4-fold reduction in IL-5, while promoting a modest increase in IL-13 concentrations in the lungs (Fig. 3D). Overall, RI-1 triggered a consistent upregulation in IL-6, IL-4, IL-13, IL-17, G-CSF and the eosinophil chemokine eotaxin and a reduction in IL-5 and IL-1β over multiple timepoints through the 4-week challenge period (Fig. S5).

In mice infected repeatedly with *A. fumigatus*, RI-1-induced changes in cytokines were accompanied by an increase in GATA3^+^ Th2 cell polarization and eosinophilia in the lungs and the circulation, despite the decrease in IL-5 (Fig. 3E and 3F). Eosinophils were identified as CD45^+^Ly6G^-^CD11c^-^SiglecF^+^CD125^+^CD64^-^ cells (Fig. S6 and S7A). Eosinophil infiltration increased with repeated fungal challenge in the chronic model and these changes were reflected when both frequencies and absolute numbers were measured (Fig. S7B). In contrast, RI-1 did not affect the abundance of the GATA3^+^ innate lymphoid cells (ILCs) suggesting that the loss of IL-5 is likely to be attributed to lower activation of type 2 cells rather than a change in cell polarization (S7C and S7D). To test whether these changes depended on NETs we administered DNase I that degrades NETs and disarms their proinflammatory activity (14). DNase I treatment suppressed the induction of IL-6 in the chronic aspergillosis model (Fig. 3G). The fungal load was not affected by RI or DNase I treatments indicating that the changes in immunological responses did not interfere with fungal control (Fig. S7E). Given the importance of IL-6 in Th17 polarization we also examined the impact of RAD51 inhibition on Th17 cell abundance and lung IL-17 cytokine concentrations (38). RI-1 increased both Th17 polarization and IL-17 concentrations in chronic aspergillosis in a NET dependent manner as indicated by their suppression upon exogenous DNase I treatment (Fig. 3G and 3H).

Another cytokine that was strongly upregulated upon RAD51 blockade was G-CSF, a critical factor that drives granulopoiesis (Fig. 3D) (39). Despite the benefits in emergency granulopoiesis, strong and sustained G-CSF induction eliminates mature neutrophils and promotes a disbalance towards immature neutrophils, a phenomenon known as neutrophil dysfunction (15). RI-1-mediated NET destabilization was accompanied by a shift towards immature neutrophils in the circulation (Fig. 3H). To confirm that these changes in the inflammatory programme depended on NETs, we infected WT animals with either a wt or a yeast-locked *Δhgc1 C. albicans* strain that does not induce NET release *in vivo* (3). RI-1 treatment boosted IL-6 production in mice infected with wt *C. albicans*, but not in animals infected with the yeast-locked *Δhgc1* mutant, showing that RI-1 did not affect inflammation in a fungal infection model where NETs were absent (Fig. 3I). These experiments indicated that the loss of NET stability was associated with dysregulation in innate and adaptive inflammatory responses. Certain cytokines such as IL-1β and IL-5 were downregulated upon RAD51 inhibition, thus exhibiting a dependence on NET-mediated stimulation, while IL-6 and its downstream target IL-17 were amplified. These changes in inflammation were accompanied by neutrophil dysfunction and eosinophilia despite the decrease in IL-5 indicating that NET destabilization promoted type-2 inflammation.

### Unstable NETs spread to the circulation and activate monocytes

**Figure 4:**
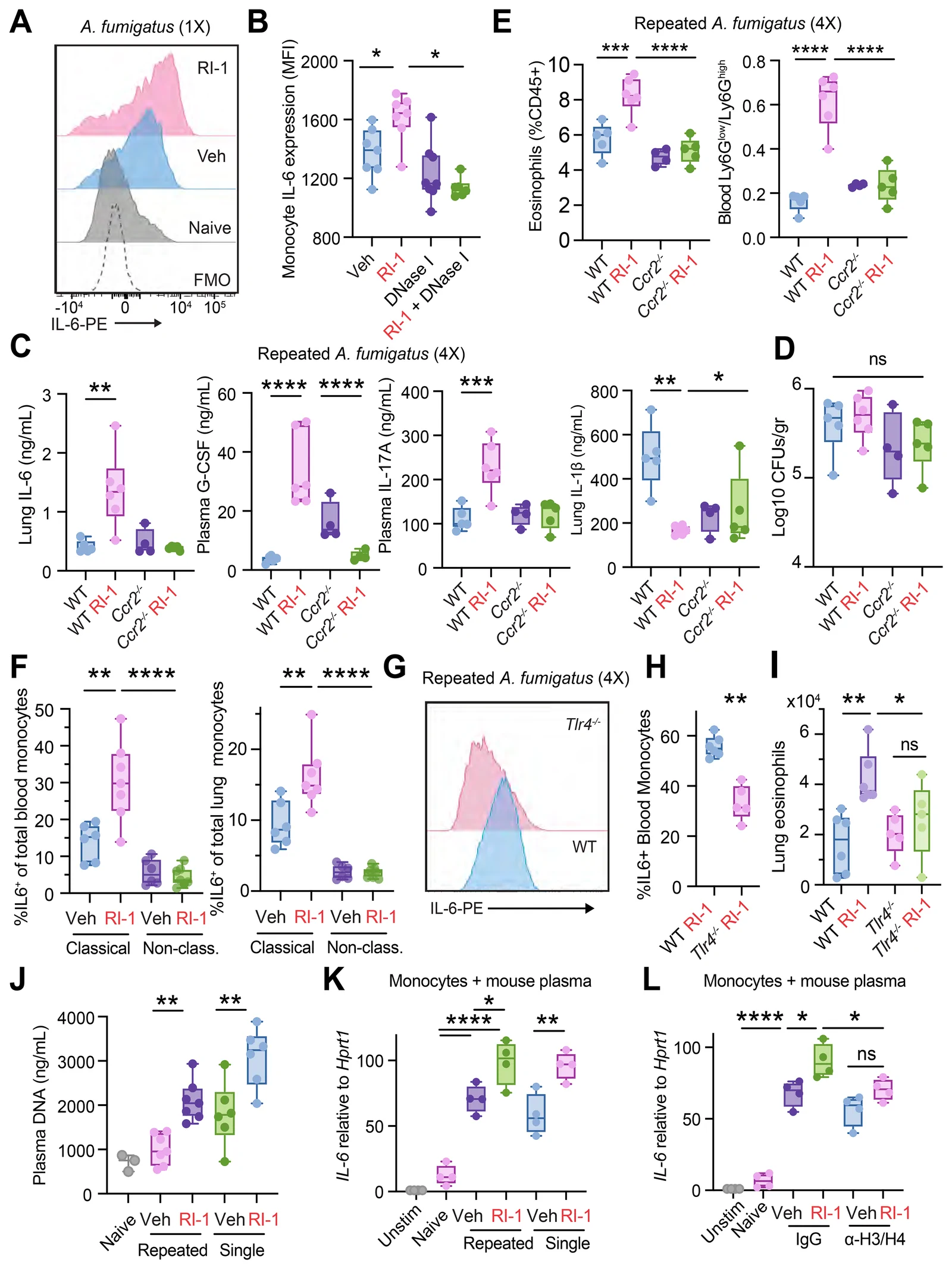
NET destabilization induces IL-6 expression in circulating monocytes (**A**) Representative flow cytometry histograms of intracellular staining for IL-6 in blood monocytes isolated from naïve mice or treated with vehicle or RI-1 and infected with A. fumigatus 24 hrs post-infection. (**B**) Quantification of mean fluorescence in tensity (MFI) of IL-6 expression in monocytes from mice treated with vehicle, RI-1, DNase I or a combination of RI1 and DNAse I, 24 hrs post-infection. (**C**) Lung and plasma cytokine concentrations (IL-1β, IL-6, G-CSF, IL-17) in WT and CCR2-deficient treated with vehicle or RI-1 and infected with 4 doses of A. fumigatus at 24 hrs after the last infection. (**D**) Lung A. fumigatus load per mouse in (C). (**E**) Lung eosinophils and blood ratio of immature over mature neutrophils in (B). (**F**) Analysis of IL-6 expression in classical and non-classical monocytes. (**G**) Intracellular IL-6 staining in blood monocytes from WT or TLR4 deficient animals treated with RI-1 24 hrs post-acute *A. fumigatus* infection. (**H**) Fraction of IL-6^+^ monocytes in (G) from 5 animals per group. (**I**) Lung eosinophils in WT or TLR4 deficient animals treated with vehicle or RI-1 24 hrs post-acute *A. fumigatus* infection. (**J**) Plasma DNA concentrations in mice treated with vehicle or RI-1 and infected with the acute or chronic A. fumigatus model, 24 hrs post-infection. (**K**) IL-6 expression relative to housekeeping *Hprt1* in human blood monocytes activated with plasma from naïve mice or treated with vehicle or RI-1 and infected with either 1 or 4 doses of A. fumigatus, 24 hrs post-infection. (**L**) As in (K) but single infection plasma was also treated with a combination of antibodies against histone H3 and H4 or control IgG antibodies. (A to L) Each data point represents an individual mouse in a single experiment, bars show the mean and 10%-90% percentile and error bars ± SEM. Representative of 3 (F and J) and 2 (B to E, K and L) independent experiments. Statistics by one-way Anova, ****p<0.0001, ***p<0.001, **p<0.01, *p<0.05, ns>0.05.

The loss of specific cytokines confirmed that NETs amplified lung inflammation as previously reported (14). However, the increases in other cytokines were more intriguing, given that the clearance of NETs would be expected to reduce inflammation evenly across all cytokines. Furthermore, IL-1β and IL-6 are typically expressed synchronously and their decoupling and opposing trends were puzzling. Therefore, we decided to investigate the link between NET destabilization and IL-6 induction, and its potential role in pathology, especially because IL-6 induces IL-4 to augment Th2 polarization (40). We investigated the cellular source of aberrant IL-6 production and found that in *A. fumigatus*-infected animals, IL-6 was strongly upregulated by RI-1 treatment in circulating monocytes in the absence of *in vitro* restimulation (Fig. 4A and 4B). DNase I treatment reversed the exacerbated IL-6 induction in circulating monocytes linking the process to NETs (Fig. 4B). To probe the contribution of monocytes to IL-6 production, we employed CCR2-deficient mice that lack circulating monocytes. Unlike WT controls, infected CCR2-deficient animals did not upregulate IL6, G-CSF or IL-17 in the lung or plasma upon RI-1 treatment despite the comparable fungal load across the groups (Fig. 4C and 4D). The RI-1-induced increase in eosinophilia and neutrophil dysfunction was absent in CCR2-deficient mice (Fig. 4E). The upregulation of IL-6 in response to RAD51 inhibition occurred in classical Ly6C^high^CCR2^high^CD43^low^ monocytes in the blood and the lungs but not in non-classical Ly6C^low^CCR2^low^CD43^high^ monocytes (Fig. 4F and S8). These experiments indicated that circulating classical monocytes were the major source of the aberrant pools of IL-6 and G-CSF.

To further investigate the dependence of RI-1-mediated cytokine dysregulation on NETs we employed *Tlr4* knockout animals, as this receptor recognises NET histones (14). IL-6 expression was reduced in monocytes derived from infected RI-1-treated *Tlr4* knockout animals (Fig. 4G and 4H). Moreover, RAD51 inhibition did not result in elevated lung eosinophilia in RI-1-treated *Tlr4* knockout animals (Fig. 4I). Hence, NET destabilization induced a dysregulated inflammatory programme in circulating monocytes that dependent on NET chromatin and TLR4.

To understand the link between NET destabilization in the lungs and monocyte activation in the circulation we measured the levels of circulating chromatin in the plasma at 24 hrs post-infection. We observed an increase in plasma cell-free DNA in RI-1 treated mice after single or repeated *A. fumigatus* challenges (Fig. 4J). Moreover, cultured human monocytes treated in vitro with plasma isolated from animals infected with *A. fumigatus* and treated with RI-1, produced higher levels of IL-6 in a manner that depended on histones as it was blocked by anti-histone antibodies (Fig. 4K and 4L). Histone blockade reversed the excess upregulation of IL-6 by RI-1 treatment without affecting the basal cytokine levels induced by plasmas from untreated infected controls that are likely to be driven by cytokines and factors other than nucleosomes. These results indicated that the faster degradation of NETs in the infected lungs, led to a higher accumulation of NET chromatin in the bloodstream that activated circulating monocytes.

### Extracellular DNA correlates with dysregulated inflammation in human aspergillosis

**Figure 5:**
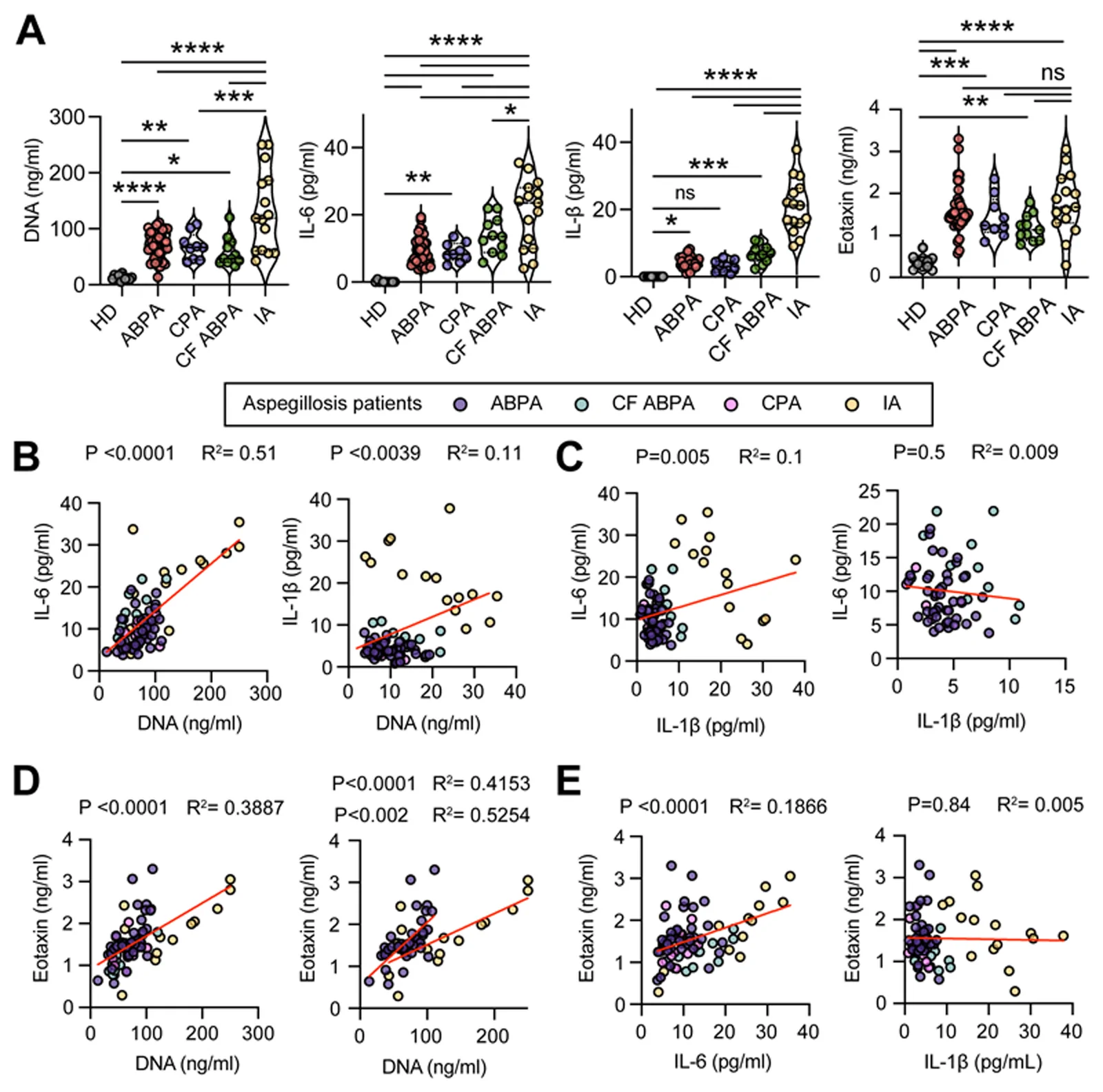
Correlation analysis of DNA and inflammatory markers in human aspergillosis (**A**) Concentrations of cell-free DNA, IL-6, IL-1β and eotaxin in the plasma of healthy control donors, or patients with allergic bronchopulmonary aspergillosis (ABPA), chronic pulmonary aspergillosis (CPA), cystic fibrosis with ABPA (CF ABPA) and invasive aspergillosis (IA). (**B** to **E**). Correlation analysis between plasma concentrations of (B) cell-free DNA and iIL-6 or IL-1β in all aspergillosis patients, (**C**) IL-6 and IL-1β in all aspergillosis patients (left) or in ABPA, CPA and CF ABPA with IA samples excluded (right), (**D**) cell-free DNA and eotaxin in all aspergillosis patients analysed collectively (left) or by comparing ABPA against IA fitted independently (right), (**E**) eotaxin and IL-6 or IL-1β in all aspergillosis patients. (A to E) Each data point represents an individual patient. Statistics by one-way Anova (A) or linear regression analysis (B to E): ****p<0.0001, ***p<0.001, **p<0.01, *p<0.05, ns>0.05.

To investigate whether chromatin-mediated immune dysregulation may be relevant in human *A. fumigatus* infections, we measured the levels of cell-free DNA, IL-6 and eotaxin in the plasmas of patients with allergic bronchopulmonary aspergillosis (ABPA), chronic pulmonary aspergillosis (CPA), cystic fibrosis with ABPA (CF ABPA) or invasive aspergillosis (IA). Compared to healthy controls donors, aspergillosis patients exhibited elevated plasma cell-free DNA, IL-6, IL-1β and eotaxin (Fig. 5A). Cell-free DNA, IL-6 and IL-1β were distinctly higher in patients with IA compared to patients with the other conditions. In contrast, the levels of eotaxin were comparably elevated across the groups.

There was a strong correlation between the concentrations of cell-free DNA and IL-6 (P<0.0001, R^2^= 0.51) within the combined aspergillosis cohort that was consistent with the dependence of IL-6 on cell-free DNA in the murine aspergillosis models (Fig. 5B). In contrast, IL-1β did not correlate with cell-free DNA. Moreover, there was no correlation between IL-1β and IL-6 within the different groups but only weakly when the IA cohort was included in the comparisons due to the overall high levels of both cytokines (Fig. 5C). Hence, the decoupling between IL-6 and IL-1β was consistent in both murine infection models and human patients.

Furthermore, cell-free DNA correlated strongly with eotaxin across the cohorts (Fig. 5D). When the ABPA and IA cohorts were assessed separately, it became evident that there was a different level of sensitivity in the induction of eotaxin with respect to the levels of cell-free DNA between the two cohorts, with ABPA reaching high eotaxin levels at a lower DNA range than IA patients. This observation explained how eotaxin could correlate with plasma DNA but be present at similar concentrations in patient groups where DNA was present at different concentration ranges. Similarly, plasma levels of IL-6 but not IL-1β, correlated well with eotaxin (Fig. 5E). These data support a role for cell-free DNA and IL-6 in promoting eosinophilia in human aspergillosis patients.

### NET destabilization promotes eosinophilia and airway obstruction via IL-6

**Figure 6:**
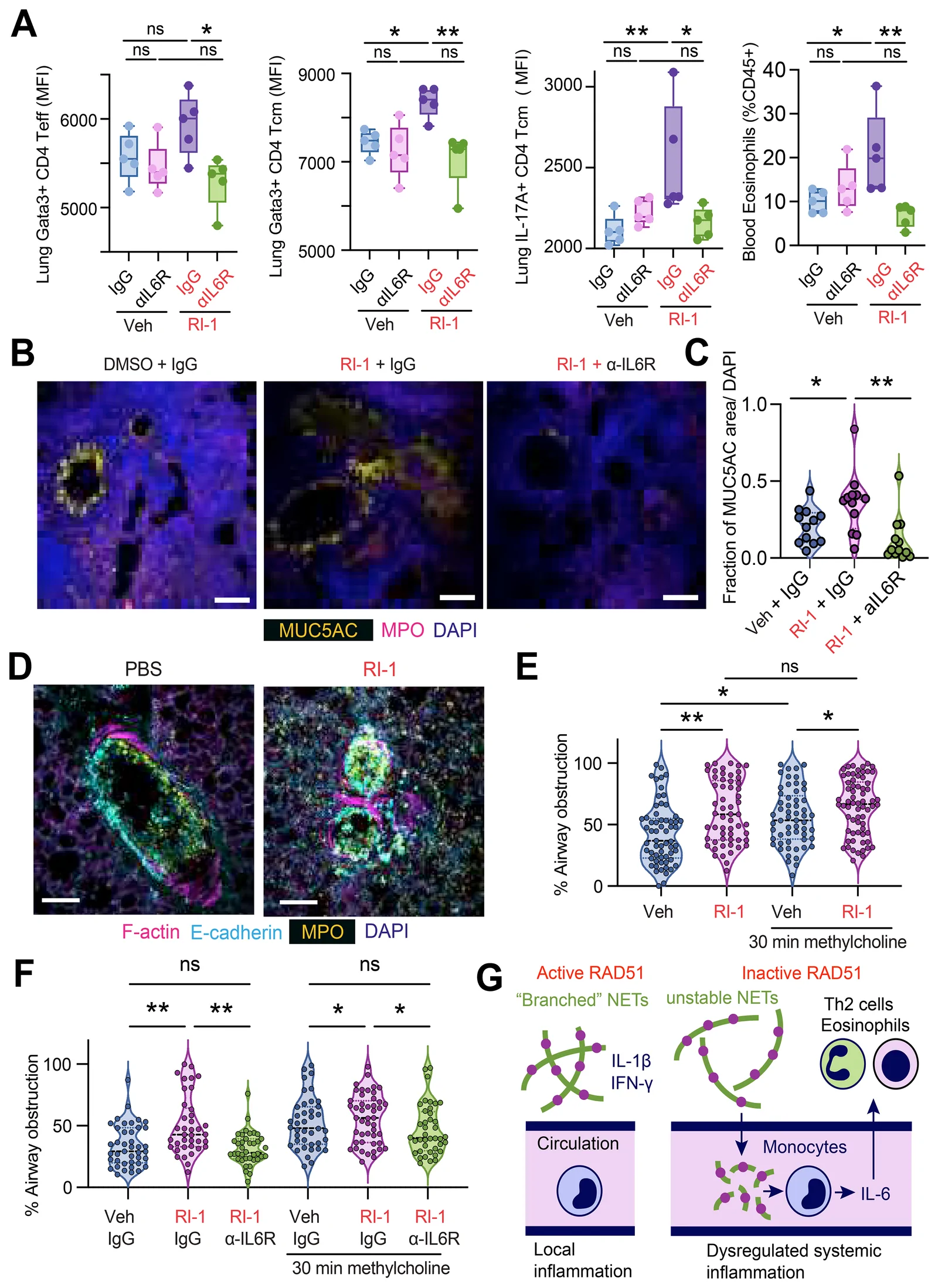
Dysregulated IL-6 drives eosinophilia and asthma (**A** to **F**). WT mice challenged with chronic pulmonary *A. fumigatus* and injected with a control IgG or anti-IL-6 blocking antibodies in the absence or presence of RI-1 treatment: (**A**) Lung Th2 T effector and memory cells, Th17 cells and eosinophils. Each data point represents an individual mouse in a single experiment, bars show the mean and 10%-90% percentile and error bars ± SEM. Representative of 2 independent experiments. (**B**) Confocal micrographs of lungs stained for MUC5AC (yellow), MPO (magenta) and DAPI (blue). Scale bars: 100 μm. (**C)** Quantification of the area of MUC5AC staining normalized to the area of DAPI. Each data point represents the percent mucin staining in one lung micrograph with 3 micrographs per animal and N=4 per group in a single experiment. Representative of 2 independent experiments. (**D**) Representative microscopy images of airway obstruction in live lung slices of WT mice treated with either vehicle or RI-1, stained for F-actin (magenta), E-cadherin (cyan) and DAPI (blue), in the absence of methylcholine. (**E** and **F**) Quantification of airway obstruction calculated from microscopy images of untreated lung slices or treated with methylcholine from two separate experiments involving RI-1 or RI-1 with control IgG or anti-IL-6 blocking antibody injections. (**G**) Model of the role of RAD51-mediated NET stabilization on the regulation of systemic inflammation. RAD51 stabilizes NETs at the sites of infection to prevent the accumulation of NET chromatin in the circulation. In the absence of RAD51-mediated NET stabilization, NET chromatin fragments accumulate in the circulation and activate monocytes to produce IL-6 that drives eosinophilia and G-CSF that promotes the surge in immature neutrophils (INs). (**E** and **F**) Each data point represents one micrograph from N=4 mice per group. Representative of 2 independent experiments. Statistics by one-way Anova: ****p<0.0001, ***p<0.001, **p<0.01, *p<0.05, ns>0.05.

Even though there is currently no direct mechanistic link between IL-6 and eosinophilia, Th17 responses that depend on IL-6 have been associated with eosinophilia in murine models of chronic aspergillosis and colitis (38, 41, 42). Therefore, we hypothesized that the unusually high levels of IL-6 may play a role in amplifying type-2 inflammation in the lungs by promoting IL-17 production to drive eosinophilia. Treatment with IL-6 receptor (IL-6R) blocking antibodies inhibited the RI-1-driven increase in Th17 and Th2 cell polarization and eosinophilia in mice (Fig. 6A). Furthermore, RI-1 treatment did not increase IL-4, IL17, eotaxin Th2 cell and eosinophil numbers in IL-6 deficient animals indicating that dysregulated Il-6 was a major driver of aberrant type-2 inflammation (Fig. S9A-D). Likewise, the lack of G-CSF upregulation in IL-6 knockouts, placed IL-6 upstream of this cytokine in this model (Fig. S9D). We noted that compared to WT controls, SiglecF^+^ macrophages were expanded in infected IL6 knockout mice but this did not depend on RI-1 treatment and did not occur in mice receiving anti-IL-6 blocking antibodies, suggesting that complete IL-6 deficiency had additional effects on macrophage diversity compared to transient blockade (Fig. S9A).

Chronic exposure to *A. fumigatus* causes a type-2 dependent obstruction of the airways affecting millions of patients (18, 43) One feature of airway hypersensitivity is augmented mucus production. RI-1 treatment increased mucus production in *A. fumigatus* infected animals in an IL6R-dependent manner, which was consistent with an increase in pathological type-2 inflammation (Fig. 6B and 6C). IL-6R blockade did not only restore mucus production to the levels of infected control animals, but reduced mucus production to near undetectable homeostatic levels. To further understand the impact of IL-6-driven aberrant type-2 inflammation on lung function, we measured airway obstruction in live lung slices isolated from mice challenged repeatedly with *A. fumigatus*, either unstimulated or after methylcholine stimulation. RI-1 increased airway obstruction both in unstimulated and methylcholine-treated lung slices (Fig. 6D and 6E). IL-6R blockade in the presence of RI-1 inhibited the increase in airway obstruction, suggesting that IL-6 was required for RI-1-mediated airway obstruction (Fig. 6F). Therefore, disrupting RAD51-mediated NET stability dysregulated inflammation during pulmonary *A. fumigatus* exposure, promoting IL-6 dependent eosinophilia that exacerbated type-2 immune pathology.

## Discussion

Our study uncovers a role for RAD51 and elements of DNA recombination in immunity through the regulation of NET stability. By interconnecting NET chromatin, RAD51 increases the structural integrity of NETs and compartmentalizes inflammation through the spatial restriction of NET-derived DAMPs. These findings suggest that ROS in NETosis may serve both as a signal that triggers NE translocation and a mediator of DNA damage that activates recombination repair processes necessary for chromatin branching. We propose that RAD51 extends the half-life of NETs and slows down the generation of mono-nucleosomes that are key proinflammatory NET components (14) and protects against the aberrant activation of monocytes in the circulation. The ability of different stimuli to generate NETs with varying degrees of stability indicates that NET architecture could be another tuneable feature that could influence disease pathogenesis. For example, the higher levels of cell-free DNA we observed in aspergillosis patients could originate from elevated NETosis, NET destabilization or defective clearance.

Unlike other cytokines, eotaxin was present at similar concentrations across human aspergillosis groups, indicating variable sensitivities to the induction of eotaxin with some groups responding to lower levels of cell-free DNA and IL-6. ABPA patients appeared more sensitized to lower levels of DNA and IL-6 than patients with invasive disease. This is likely due to differences in adaptive immune responses. ABPA is a chronic condition involving long-term adaptive Th17 and Th2 conditioning that amplifies responses to inflammatory cues. In contrast, invasive aspergillosis patients are under immune suppression, which may reduce DNA and IL-6-mediated signalling.

Our findings shed light into the critical role for NET stability in shaping the local and systemic induction of inflammatory cytokines. NET destabilisation led to an unusual decoupling between IL-1β and IL-6, with a similar trend in human aspergillosis plasmas. Generally, these cytokines exhibit similar expression patterns particularly in NET-driven inflammation (13, 44). The reduction in IL-1β under conditions where IL-6 was amplified was likely due to differences in the regulation of these cytokines. Il-1β requires priming and inflammasome activation whereas Il-6 secretion is independent of inflammasome activation (45). NETs are potent priming signals that induce the transcription of these cytokines, but are poor inflammasome activators (13). NET-mediated priming of monocytes in the circulation in the likely absence of inflammasome-activating signals was sufficient to drive IL-6 but not IL-1β secretion. In contrast, the accelerated degradation of NETs in the lungs where microbial inflammasome activators such as fungal hyphae were present lowered IL-1β secretion (44, 46). Hence, the location of immune activation can influence the repertoire of secreted cytokines.

The induction of aberrant IL-6 caused by unstable NETs drove an IL-6-dependent pathway that augmented Th2 and Th17 responses and drove eosinophilia, despite the reduction in IL-5. Hence, high IL-6 levels could override the requirement for IL-5, which is central to eosinophil recruitment (47). Our results are in line with a previously reported link between pathogenic eosinophilia and Th17 dysregulation in repeated *A. fumigatus* and murine colitis models and the established role of IL-6 in augmenting Th2 cell polarization (40, 41). Moreover, dual-positive Th2/Th17 activation has been reported in patients with late-onset eosinophilic asthma (48). The dysregulated eosinophilia in the context of NET destabilization was also consistent with the different roles of IL-1β and IL-6 in coordinating Th17 responses. IL-6 is essential for Th17 differentiation whereas IL-1β is an amplifier of Th17 responses (38).

The role of RAD51 in promoting NET stability may be relevant in many inflammatory contexts including cancer. DNA repair pathway inhibitors are used in the treatment of many cancers to promote genomic instability in tumours and boost antigen presentation (49, 50). NETs have been found to be pathogenic in cancer by promoting metastasis, reactivating dormant tumours and interfering with cancer immunotherapies (51). Based on these findings, the therapeutic benefits of targeting DNA repair could also implicate the destabilization of NETs in the tumour microenvironment (52, 53).

While our study implicates DNA recombination in NET biology and the regulation of inflammation, it opens additional mechanistic questions. Although RAD51 was implicated in NET branching, the mechanistic details of this process will be important to unveil in future studies. It is likely that in the absence of resolvase-mediated DNA incisions, RAD51-mediated NET branches remain unresolved. Moreover, RAD51 may promote DNA co-aggregation as it scans for homologous DNA sequences without forming HJs. Furthermore, despite NET branching reduction upon RAD51 inhibition, other mechanisms of DNA repair, or completely unconventional mechanisms involving granule proteins (8, 12, 54).

Eosinophilia is found in roughly half of all asthma cases (55). Likewise, fungal colonization is encountered in a substantial fraction of asthma patients and several studies have demonstrated the effectiveness of antifungals in asthma management (43, 56). Despite recent advances in asthma management, eosinophilic or mixed granulocytic asthma remain difficult to treat (57, 58). Recent studies have demonstrated elevated IL-6 concentration in patients with granulocytic asthma (59). Moreover, IL-6 correlates with poor lung function in asthma patients and exhibits a substantial link with obesity, a condition that can also elevate NETosis (13, 60). Our findings in mice and humans further support a role for IL-6 in promoting asthma pathology and highlight the potential for NET stabilization to regulate disease.

## Materials and Methods

### Animals

All mice were bred and maintained under specific-pathogen–free conditions on a 12 h light–dark cycle. Experiments were performed with age- and sex-matched, cage-controlled, 8–16-week-old wild-type C57BL/6J and CCR2^−^/^−^ mice, in accordance with the Francis Crick institute guidelines and UK Home Office regulations under the Animals (Scientific Procedures) Act 1986 (ASPA). Mixed sexes were used in all experiments. Breeding and experimental protocols were approved by the Francis Crick Institute AWERB sub-committee and the Home Office under project licenses with PPL numbers: 700881 granted 1 Nov 2015, PP0858308, granted on 21 Oct 2020 and PP3675387 granted 25 Nov. 2025. Mice were infected with *Candida albicans* (SC5314) or *Aspergillus fumigatus* (13073). Animals were euthanized using approved schedule 1K methods. Experiments were designed based on prior pilot studies and 90% power calculations.

### Murine infection models

For pulmonary *A. fumigatus* infection, strain 13073 was cultured on Sabouraud dextrose agar (SDA) at 37 °C for 3 days. Mice were infected intratracheally with 1 × 10^7^ swollen conidia in PBS. For acute infection, animals were sacrificed 24 h post-inoculation. For the chronic infection, mice received intratracheal inoculations every 7 days for 4 weeks and were sacrificed 24 h after the final challenge. For pulmonary *C. albicans* infection, wild-type SC5314 or yeast-locked *hgc1Δ C. albicans* were cultured overnight at 37 °C in YEPD (Sigma) with shaking, and sub-cultured for 4 h to an OD_600 of 0.4–0.8. Mice were then infected intratracheally with 2 × 10^6^ *C. albicans* in PBS. Where indicated, mice received intraperitoneal RI-1 (1 mg), DNase I (2000 U/mouse), both treatments, anti-IL-6R antibody (250 μg) or rat IgG2b isotype control on day −1, day 0 and day +1 relative to infection.

### Lung fungal burden

Lung lobes were weighed, homogenized in PBS, serially diluted and plated on SDA supplemented with 100 µg/ml streptomycin to prevent bacterial contamination. Plates were incubated at 37 °C for 12–18 h, and colony-forming units (CFUs) were enumerated and normalized to lung weight.

### Cytokine quantification in lung and plasma

Lung lysates were homogenised in lysis buffer (PBS containing 0.5% Triton X-100, 1× cOmplete protease inhibitor, and 1× PhosSTOP; Sigma-Aldrich). Protein concentrations were determined using the Pierce BCA Protein Assay Kit (Thermo Scientific). Cytokines in lung lysates and plasma were measured using Bio-Plex Pro Mouse Cytokine Assays and analysed with a Luminex Bio-Plex 200 system (Bio-Rad).

### Flow cytometry

Mice were euthanized and perfused with PBS via the right ventricle. Lungs were minced and digested in Liberase TL (0.2 mg/ml; Roche) and DNase I (0.1 mg/ml; Roche) at 37 °C for 1 h with shaking. Homogenates were passed through a 70 µm filter and centrifuged at 300 × g for 10 min. Whole blood was centrifuged at 300 × g for 10 min to separate leukocytes and plasma. Red blood cell lysis was performed with ACK buffer (Gibco). Single-cell suspensions were incubated with anti-FcγRIII/II (Fc block; BD Pharmingen) for 30 min, stained with LIVE/DEAD™ Fixable Blue (Thermo Fisher), fixed with the BD Transcription Factor Phospho Buffer Set (BD Biosciences), and labelled with fluorochrome-conjugated antibodies (Supplementary Table 1). Samples were acquired on a Cytek Aurora and analyzed using FlowJo software.

### Human blood immune cell isolation

Peripheral blood was obtained from healthy adult volunteers with informed consent, in compliance with the Francis Crick Institute ethics board and the UK Human Tissue Act. Blood collected in EDTA tubes was layered on Histopaque-1119 (Sigma-Aldrich) and centrifuged at 800 × g for 20 min to separate plasma, PBMC, and neutrophil fractions.

### In vitro stimulation of human monocytes

CD14^+^ monocytes were purified from PBMCs using MACS CD14 microbeads (Miltenyi Biotec). Cells were cultured in RPMI (Gibco) supplemented with 1% L-glutamine, 100 U/ml penicillin, and 100 µg/ml streptomycin, and stimulated with 3% plasma from naïve or infected mice (± RI-1 treatment) for 16 h at 37 °C. Where indicated, plasma was pre-incubated with anti-histone H3 and H4 antibodies (Millipore) or control rabbit IgG (BioXCell) for 1 h at 37 °C. All stimulants were pretreated with 50 µg/ml polymyxin B (Invivogen) to neutralize endotoxin. Total RNA was extracted using TriReagent/Chloroform/Isopropanol (Sigma-Aldrich). cDNA was synthesized from 2 µg RNA with the Transcriptor High Fidelity cDNA Synthesis Kit (Roche) using anchored-oligo(dT)_18 primers. *Il6* expression was quantified by qPCR using TaqMan Universal PCR Master Mix (Applied Biosystems) on a 7900HT Fast Real-Time PCR System, normalized to HPRT1 and calculated using the ΔΔCT method.

### Hoxb8 cell culture and RAD51 genetic manipulation

Murine Hoxb8 cells generated in the lab were cultured in RPMI medium supplemented with L-glutamine, FBS, penicillin–streptomycin, β-estradiol (1μM), and CHO-SCF cell line-conditioned medium (2.5%). Cells were passaged every 3-4 days. To differentiate Hoxb8 cells into neutrophils, the medium was replaced with RPMI containing L-glutamine, FBS, penicillin–streptomycin, CHO-SCF conditioned medium, and mouse G-CSF (20ng/ml) for 5 days. Silencing of RAD51 in Hoxb8 cells was performed using ShRNA technology. The sequences were inserted in a PGK-EGFP-tetR backbone. Sequences used were: Sh1: CCTGTGATGCTATACGGCTTT, Sh2: CGGTCAGAGATCATACAGATA, Scramble: CCTAAGGTTAAGTCGCCCTCG. Cells were stably infected with retroviruses carrying Sh1, Sh2, or scrambled plasmids, sorted for GFP expression, and cultured for 48 h with 2μg/ml doxycycline on day 3 upon differentiation. Total cells and viable cells were measured with a Vi-CELL BLU (Beckman Coulter) counter.

### Histology and immunofluorescence imaging

Mouse lungs were fixed directly in 10% formalin for 24 h, transferred to 70% ethanol for 24h, and embedded in paraffin. 4μm sections were cut with a standard microtome and placed onto positively charged glass slides. Sections were baked at 60°C for 1h, deparaffinised in three sequential 5-min baths of Neo-Clear, and rehydrated through graded ethanol baths (100%, 96%, 80%, 70%, and 50%; 5 min each), followed by washing. Antigen retrieval was performed using Dako Target Retrieval Solution (pH 9) for 45 min at 97°C. Sections were permeabilised in 0.5% Triton X-100 in PBS for 5 min at room temperature. Non-specific binding was blocked in 2% BSA (Sigma) and 2% donkey serum (Sigma) in PBS for 1h at RT. Slides were incubated overnight in a humidified chamber with primary antibodies diluted in blocking buffer (Supplementary Table 1). Sections were then washed in PBS and incubated for 1h at RT in a humidified dark chamber with labelled secondary antibodies (Supplementary Table 1). Stained sections were mounted in ProLong Gold (Molecular Probes). Images were acquired using a Leica TCS SP8 inverted confocal microscope (20× or 40× magnification) and analysed in Fiji/ImageJ.

### Precision-cut ex vivo lung slices

24 h after the final intratracheal infection with *A. fumigatus*, mice were euthanised by intraperitoneal pentobarbital injection, with death confirmed by exsanguination via the femoral artery. Lungs were inflated with 2% low-melting agarose (pre-warmed to 37°C) in HBSS++ via insertion of a 20 G venous catheter into the trachea. Ice was applied externally until the agarose solidified. Lobes were then separated, washed in PBS, and incubated overnight at 37°C and 5% CO_2_ in DMEM/F-12 supplemented with 10% FBS and penicillin–streptomycin. The following day, 200μm lung slices were cut on a Leica VT1200 S vibratome and incubated overnight at 37°C and 5% CO_2_ in DMEM/F-12 with 10% FBS and penicillin–streptomycin.

### Methylcholine treatment and staining of lung slices

Lung slices were treated with 500 mg/ml methylcholine (acetyl-β-methylcholine chloride; Sigma A2251) in HBSS with Ca^2+^ and Mg^2+^ for 30 min at 37°C and 5% CO_2_. Methylcholine is a non-specific muscarinic receptor agonist used to study airway hyperresponsiveness in asthma. Slices were then washed with PBS and fixed in 4% PFA for 15 min at RT. Sections were permeabilised in 0.5% Triton X-100 in PBS for 5 min at RT and washed in PBS. Non-specific binding was blocked with 2% BSA (Sigma) and 2% donkey serum (Sigma) in PBS for 1h at RT. Samples were incubated overnight in a humidified chamber with anti-E-cadherin antibody (BD Biosciences, 610181). The following day, slices were incubated for 1h at RT in a humidified dark chamber with donkey anti-mouse 488 antibody (A21092) in blocking buffer. Actin (Phalloidin, A12380) and nuclear (DAPI; Invitrogen) dyes were added during the secondary antibody incubation. All sections were mounted in ProLong Gold (Molecular Probes) on glass slides. Images were taken on a Leica SP8 inverted confocal microscope (20× magnification) and analysed manually in Fiji/ImageJ. Free lumen area was normalised to total lumen area to assess airway occlusion.

### Human neutrophil isolation

The neutrophil fraction collected from whole blood was washed in HBSS without Ca2+ and Mg2+ containing 0.1% FBS. Neutrophils were purified on a discontinuous Percoll gradient (GE Healthcare) composed of 1.105 g/ml (85%), 1.100 g/ml (80%), 1.093 g/ml (75%), 1.087 g/ml (70%), and 1.081 g/ml (65%) layers, and centrifuged at 800 x g for 20min. Neutrophil-enriched fractions were collected and washed once before use.

### Stimulation of human neutrophils

1 × 10^6^ neutrophils were seeded on glass coverslips in 24-well plates in HBSS containing Ca2+ and Mg2+, supplemented with 100 mM HEPES and 3% autologous plasma. Cells were incubated for 30 min at 37°C and 5% CO_2_, then stimulated with PMA (100 nM), neutrophil elastase inhibitor (100 μM), or ionomycin (1 μM) for 90 min, or with heat-inactivated *C. albicans* hyphae (5 × 10^6^ per well) for 4 h.

### Staining and imaging of human neutrophils

DNA strand breaks in neutrophils were detected using the Click-iT™ TUNEL Alexa Fluor™ Imaging Assay (C10246) according to the manufacturer’s instructions. Primary antibodies included RAD51 (Abcam, ab213) and neutrophil elastase (GeneTex, GTX72042). Imaging was performed using a Leica TCS SP5 inverted confocal microscope (20× magnification).

### NET degradation by gel electrophoresis

Neutrophils were seeded at a density of 1 × 10^6^ cells per well in 12-well plates in HBSS++ supplemented with 10 mM HEPES and either 50 µM RI-1 or DMSO (vehicle control). The medium was equilibrated to 37 °C prior to cell addition. Cells were allowed to settle and adhere for 45 min at 37 °C. NET formation was induced with 100 nM phorbol 12-myristate 13-acetate (PMA) and incubated overnight at 37 °C. DNase I prepared in pre-warmed buffer and added at final concentrations of 5 U/mL, 0.5 U/mL, and 0.05 U/mL for 20 min at 37 °C. Supernatants were collected and transferred to Eppendorf tubes containing 0.5 M EDTA, then centrifuged for 10 min at room temperature, and either stored at −20 °C or analysed immediately using a 0.8% agarose gel. Electrophoresis was performed for 30 min at 120 V, and gels were imaged immediately thereafter.

### NET degradation timelapse assay for murine Hoxb8-derived neutrophils

5 × 10^4^ Hoxb8-derived neutrophils were seeded in black 96-well plates (PerkinElmer) in HBSS containing Ca^2+^ and Mg^2+^, 0.5% CHO-SCF, 0.2μM Sytox Green (membrane-impermeable, to stain dead cells; Invitrogen), and 4μg/ml Hoechst (membrane-permeable, to stain live cells; Thermo Scientific). Cells were incubated for 45 min at 37°C and 5% CO_2_ before stimulation with 100nM PMA (Sigma). Imaging was performed on an inverted Nikon wide-field microscope system at 37°C and 5% CO_2_. 4 fields of view were acquired per well every 30 min for 24 h. NET degradation was quantified fusing ImageJ from timelapse movies depicting an expansion of nuclear area during NETosis and a subsequent loss of nuclear area and shrinkage as NETs degraded. Individual NETs were identified as DNA objects that expanded to an excess of 200 μm^2^. The maximal area for each object was recorded and then DNA area loss was tracked at 1 hr intervals during the timelapse. These traces were fitted using Graph Pad prism to generate decay curves and calculate the half-life for the NETs in each condition.

### Human blood neutrophil-derived NET degradation by time-lapse microscopy

In (Fig. 1H and S3D to F) NET degradation was quantified from time-lapse movies using ImageJ software. Regions of interest (ROIs) corresponding to individual neutrophils were identified by thresholding the Hoechst channel, followed by measuring the Sytox signal. Each field of view contained both NETotic and necrotic cells. NETotic were distinguished from necrotic cells by analysing Sytox intensity traces: continuously increasing Sytox intensity with maximum values occurring within the final 10% of the experiment was classified as necrotic; NETotic cells were defined as those reaching maximum Sytox intensity within the first 25% of the acquisition time after stimulation, followed by a decline in signal using a script (20, 61). In (Fig. 1K, 1L and 2B to D) NETs were identified based on their size by creating ROIs selected for extracellular chromatin that exceeded 500 μm^2^ at 8 hrs post-stimulation. Changes in mean Sytox fluorescence intensity were measured for every frame for each NET and traces were combined for all NETs per timelapse movie. Multiple movies from 2-3 independent replicates were used per condition to calculate the change in mean NET Sytox fluorescence intensity over time. Traces of mean NET formation and degradation per movie were generated and compared across multiple movies by two-way Anova. In addition, the decrease in mean NET fluorescence at endpoint from the maximum cumulative NET fluorescence of each movie was calculated and plotted for each condition. Cumulative NET half-lives were also calculated based on the time required for 50% loss in NET fluorescence intensity.

### Human aspergillosis patient studies

Samples from patients with chronic respiratory fungal diseases were collected through TrIFIC: Targeting Immunotherapy for Fungal Infections in Cystic Fibrosis (IRAS ID: 270828; REC reference: 20/LO/0110). The studies were conducted in accordance with the recommendations for physicians involved in research on human subjects adopted by the 18th World Medical Assembly, Helsinki 1964, and later revisions. All patients undergoing a symptom-driven bronchoscopy as part of routine clinical care at a single centre between January and September 2021 were approached to take part in the Lung Transplant AspiCLAD study. Ethical approval was obtained under two separate biobank applications (REC:23/EM/009; REC:21/PR/0981). All patients provided written informed consent prior to participation in the study. Blood was taken at the time of the clinical bronchoscopy, plasma was obtained and stored at -80°C until analysis. Infection status was confirmed through standard clinical microbial screening of bronchoalveolar lavage fluid.

### Western immunoblotting of human blood neutrophils and Hoxb8 cell lysates

Neutrophils (1×10^6^) were plated in 6 well plates for each condition and lysed with 500μL of 1X SDS buffer and resolved in Any kD precast polyacrylamide gel (Biorad). Proteins were transferred onto PVDF membrane, blocked for 1h with 5% milk and incubated in primary antibody (Supplementary Table 1) in 2.5% milk overnight, followed by incubation with secondary antibody (anti-mouse HRP Cat. 31455). Membranes were developed either onto film in dark room or using Biorad ChemiDoc.

### CRISPR–Cas9 editing of Rad51 in HoxB8 cells

CRISPR–Cas9 ribonucleoprotein (RNP) complexes were assembled using recombinant Cas9 (IDT; 12.5 μM) and synthetic sgRNA (Synthego) targeting murine Rad51 (5′-GCGCATATGCTACATTATCT-3′). RNPs were formed at a 3:1 sgRNA: Cas9 molar ratio and incubated at room temperature for 10 min. HoxB8 progenitor cells (1.5 × 10^5^ per reaction) were washed in PBS, resuspended in Neon buffer R, and electroporated with RNP complexes using a Neon Transfection System (1550 V, 10 ms, 3 pulses). Cells were immediately transferred to pre-warmed medium in 24-well plates and cultured under standard conditions with media changes every 3–7 days. Editing efficiency was determined by PCR amplification of the target locus followed by mismatch cleavage analysis (GeneArt Genomic Cleavage Detection Kit, Thermo Fisher Scientific). For clonal isolation, cells were subjected to limiting dilution and expanded for 2–3 weeks prior to screening by immunoblotting.

### RAD51 strand exchange assay

DNA strand exchange assays were carried out between ϕX174 virion ssDNA and linearised ϕX174 dsDNA. Reactions (10 µl) were performed in a staged manner. Firstly, RAD51 (10 μM) was incubated with ϕX174 ssDNA (30 μM, nucleotides) in reaction buffer (40 mM Tris–HCl, pH 8.0, 2 mM ATP, 1 mM MgCl_2_, 1 mM TCEP) for 5 min at 37°C. The reactions were then supplemented with (NH_4_)_2_SO_4_ (150 mM) and RPA (1 μM), and incubation was continued for 5 min. ApaLI-linearised ϕX174 dsDNA (10 μM) was then added and incubation continued at 37°C for 1 hour. Samples were deproteinized by addition of 2 μl of 5× stop buffer (100 mM Tris–HCl, pH 8.0, 10 mg/ml proteinase K, 2.5% (w/v) SDS) and incubated at 37°C for 10 min. Reaction products were separated on a 0.9% agarose /TAE gel and visualised by ethidium bromide staining. Where indicated, the RAD51 inhibitor R1-1 was added at the start of the reaction. DMSO was used as a control.

## Supplementary Material

Supplementary Materials

## Data Availability

All data needed to evaluate the conclusions in the paper are available in the manuscript or the supplementary materials. A script for automatic NET identification that was used in experiments in Fig. 1G, 1H and Fig. S3D-F (20, 61). Retroviral vectors for RAD51 knockdowns generated in this study can be provided upon request.

## References

[1] Brinkmann V (2004). Neutrophil extracellular traps kill bacteria. Science.

[2] Kenny EF (2017). Diverse stimuli engage different neutrophil extracellular trap pathways. Elife.

[3] Branzk N (2014). Neutrophils sense microbe size and selectively release neutrophil extracellular traps in response to large pathogens. Nat Immunol.

[4] Bouchery T (2020). Hookworms Evade Host Immunity by Secreting a Deoxyribonuclease to Degrade Neutrophil Extracellular Traps. Cell Host Microbe.

[5] Thanabalasuriar A (2019). Neutrophil Extracellular Traps Confine Pseudomonas aeruginosa Ocular Biofilms and Restrict Brain Invasion. Cell Host Microbe.

[6] Papayannopoulos V (2018). Neutrophil extracellular traps in immunity and disease. Nature reviews Immunology.

[7] Hidalgo A (2022). Neutrophil extracellular traps: from physiology to pathology. Cardiovasc Res.

[8] Papayannopoulos V, Metzler KD, Hakkim A, Zychlinsky A (2010). Neutrophil elastase and myeloperoxidase regulate the formation of neutrophil extracellular traps. Journal of Cell Biology.

[9] Martinod K (2013). Neutrophil histone modification by peptidylarginine deiminase 4 is critical for deep vein thrombosis in mice. Proc Natl Acad Sci U S A.

[10] Wang Y (2009). Histone hypercitrullination mediates chromatin decondensation and neutrophil extracellular trap formation. J Cell Biol.

[11] Bertran MT (2024). A cyclic peptide toolkit reveals mechanistic principles of peptidylarginine deiminase IV regulation. Nature communications.

[12] Burn GL (2025). Myeloperoxidase transforms chromatin into neutrophil extracellular traps. Nature.

[13] Warnatsch A, Ioannou M, Wang Q, Papayannopoulos V (2015). Inflammation. Neutrophil extracellular traps license macrophages for cytokine production in atherosclerosis. Science.

[14] Tsourouktsoglou TD (2020). Histones, DNA, and Citrullination Promote Neutrophil Extracellular Trap Inflammation by Regulating the Localization and Activation of TLR4. Cell reports.

[15] Ioannou M (2022). Microbe capture by splenic macrophages triggers sepsis via T cell-death-dependent neutrophil lifespan shortening. Nature communications.

[16] Toussaint M (2017). Host DNA released by NETosis promotes rhinovirus-induced type-2 allergic asthma exacerbation. Nat Med.

[17] Denning DW (2024). Global incidence and mortality of severe fungal disease. Lancet Infect Dis.

[18] Shreiner AB (2012). Repeated exposure to Aspergillus fumigatus conidia results in CD4+ T cell-dependent and -independent pulmonary arterial remodeling in a mixed Th1/Th2/Th17 microenvironment that requires interleukin-4 (IL-4) and IL-10. Infect Immun.

[19] Jimenez-Alcazar M (2017). Host DNases prevent vascular occlusion by neutrophil extracellular traps. Science.

[20] Aramburu IV (2022). Functional proteomic profiling links deficient DNA clearance with increased mortality in individuals with severe COVID-19 pneumonia. Immunity.

[21] Jimenez-Alcazar M (2015). Impaired DNase1-mediated degradation of neutrophil extracellular traps is associated with acute thrombotic microangiopathies. J Thromb Haemost.

[22] Kenny EF, Raupach B, Abu Abed U, Brinkmann V, Zychlinsky A (2019). Dnase1-deficient mice spontaneously develop a systemic lupus erythematosus-like disease. Eur J Immunol.

[23] Hakkim A (2010). Impairment of neutrophil extracellular trap degradation is associated with lupus nephritis. Proceedings of the National Academy of Sciences of the United States of America.

[24] Dhawan UK (2021). Hypercholesterolemia Impairs Clearance of Neutrophil Extracellular Traps and Promotes Inflammation and Atherosclerotic Plaque Progression. Arteriosclerosis, thrombosis, and vascular biology.

[25] Metzler KD, Goosmann C, Lubojemska A, Zychlinsky A, Papayannopoulos V (2014). A myeloperoxidase-containing complex regulates neutrophil elastase release and actin dynamics during NETosis. Cell reports.

[26] Fuchs TA (2007). Novel cell death program leads to neutrophil extracellular traps. J Cell Biol.

[27] Harbort CJ (2015). Neutrophil oxidative burst activates ATM to regulate cytokine production and apoptosis. Blood.

[28] Bonilla B, Hengel SR, Grundy MK, Bernstein KA (2020). RAD51 Gene Family Structure and Function. Annu Rev Genet.

[29] Baumann P, Benson FE, West SC (1996). Human Rad51 protein promotes ATP-dependent homologous pairing and strand transfer reactions in vitro. Cell.

[30] Sung P, Robberson DL (1995). DNA strand exchange mediated by a RAD51-ssDNA nucleoprotein filament with polarity opposite to that of RecA. Cell.

[31] Chen Z, Yang H, Pavletich NP (2008). Mechanism of homologous recombination from the RecA-ssDNA/dsDNA structures. Nature.

[32] Garcin EB (2019). Differential Requirements for the RAD51 Paralogs in Genome Repair and Maintenance in Human Cells. PLoS Genet.

[33] Song Q, Hu Y, Yin A, Wang H, Yin Q (2022). DNA Holliday Junction: History, Regulation and Bioactivity. Int J Mol Sci.

[34] Wyatt HD, West SC (2014). Holliday junction resolvases. Cold Spring Harbor perspectives in biology.

[35] Budke B (2012). RI-1: a chemical inhibitor of RAD51 that disrupts homologous recombination in human cells. Nucleic Acids Res.

[36] Shkundina IS, Gall AA, Dick A, Cocklin S, Mazin AV (2021). New RAD51 Inhibitors to Target Homologous Recombination in Human Cells. Genes (Basel).

[37] Wang GG (2006). Quantitative production of macrophages or neutrophils ex vivo using conditional Hoxb8. Nat Methods.

[38] Veldhoen M, Hocking RJ, Atkins CJ, Locksley RM, Stockinger B (2006). TGFbeta in the context of an inflammatory cytokine milieu supports de novo differentiation of IL-17-producing T cells. Immunity.

[39] Manz MG, Boettcher S (2014). Emergency granulopoiesis. Nature reviews Immunology.

[40] Rincon M, Anguita J, Nakamura T, Fikrig E, Flavell RA (1997). Interleukin (IL)-6 directs the differentiation of IL-4-producing CD4+ T cells. J Exp Med.

[41] Griseri T (2015). Granulocyte Macrophage Colony-Stimulating Factor-Activated Eosinophils Promote Interleukin-23 Driven Chronic Colitis. Immunity.

[42] Murdock BJ (2012). Interleukin-17 drives pulmonary eosinophilia following repeated exposure to Aspergillus fumigatus conidia. Infect Immun.

[43] Denning DW (2014). Fungal allergy in asthma-state of the art and research needs. Clin Transl Allergy.

[44] Warnatsch A (2017). Reactive Oxygen Species Localization Programs Inflammation to Clear Microbes of Different Size. Immunity.

[45] Broz P, Dixit VM (2016). Inflammasomes: mechanism of assembly, regulation and signalling. Nature reviews Immunology.

[46] Cheng SC (2011). The dectin-1/inflammasome pathway is responsible for the induction of protective T-helper 17 responses that discriminate between yeasts and hyphae of Candida albicans. J Leukoc Biol.

[47] Buchheit KM (2024). Interleukin-5 as a pleiotropic cytokine orchestrating airway type 2 inflammation: Effects on and beyond eosinophils. Allergy.

[48] Irvin C (2014). Increased frequency of dual-positive TH2/TH17 cells in bronchoalveolar lavage fluid characterizes a population of patients with severe asthma. The Journal of allergy and clinical immunology.

[49] Li LY, Guan YD, Chen XS, Yang JM, Cheng Y (2020). DNA Repair Pathways in Cancer Therapy and Resistance. Front Pharmacol.

[50] Carlsen L, El-Deiry WS (2022). Anti-cancer immune responses to DNA damage response inhibitors: Molecular mechanisms and progress toward clinical translation. Front Oncol.

[51] Adrover JM, McDowell SAC, He XY, Quail DF, Egeblad M (2023). NETworking with cancer: The bidirectional interplay between cancer and neutrophil extracellular traps. Cancer cell.

[52] Grecian R, Whyte MKB, Walmsley SR (2018). The role of neutrophils in cancer. Br Med Bull.

[53] Hsu BE (2019). Immature Low-Density Neutrophils Exhibit Metabolic Flexibility that Facilitates Breast Cancer Liver Metastasis. Cell reports.

[54] Metzler KD (2011). Myeloperoxidase is required for neutrophil extracellular trap formation: implications for innate immunity. Blood.

[55] Carr TF, Zeki AA, Kraft M (2018). Eosinophilic and Noneosinophilic Asthma. American journal of respiratory and critical care medicine.

[56] Rapeport WG, Ito K, Denning DW (2020). The role of antifungals in the management of patients with severe asthma. Clin Transl Allergy.

[57] Walford HH, Doherty TA (2014). Diagnosis and management of eosinophilic asthma: a US perspective. J Asthma Allergy.

[58] Dhariwal J (2021). Real-World Effectiveness of Anti-IL-5/5R Therapy in Severe Atopic Eosinophilic Asthma with Fungal Sensitization. J Allergy Clin Immunol Pract.

[59] Chu DK (2015). Therapeutic potential of anti-IL-6 therapies for granulocytic airway inflammation in asthma. Allergy Asthma Clin Immunol.

[60] Adair D (2025). High Interleukin (IL)-6 is Associated with Lower Lung Function and Increased Likelihood of Metabolic Dysfunction in Asthma. Pulm Ther.

[61] Aramburu IV (2022). Code for the NETase values in Aramburu IV et al 2022 Immunity.

