# Supplementary material for: RAD51 stabilizes neutrophil extracellular traps to compartmentalize inflammation
Source: Science. Author manuscript; Available in PMC 2026 Sep 4. (PMC7619443; doi:10.1126/science.aed9286)
Supplement: Supplementary file 1 [file EMS218022-supplement-Supplementary_Materials.pdf]

Science
AAAS

Supplementary Materials for

RAD51 stabilizes neutrophil extracellular traps to compartmentalize inflammation

Lorenza Iolanda Tsansizi, Sophie Yihan Guan1, Iker Valle Aramburu, Rajvee Shah Punatar, Thomas J. Williams, Yihe E. Qiao, Anna Reed, Darius Armstrong-James, Stephen C. West, Venizelos Papayannopoulos

Correspondence to: veni.p@crick.ac.uk

This PDF file includes:

Figs. S1 to S9
Table S1

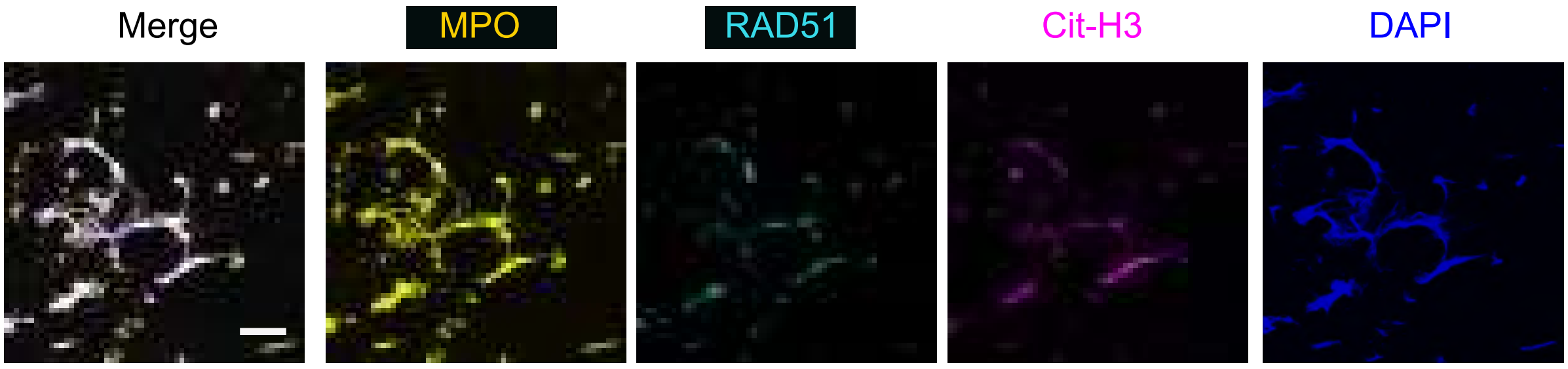

Fig. S1. RAD51 associates with NET DNA *in vivo*.
Fluorescence microscopy images of mouse lungs 24 hrs post-fungal infection, stained for RAD51 (magenta), NE (yellow), MPO (cyan), and DNA (DAPI, blue). Scale bar: 40 µm.

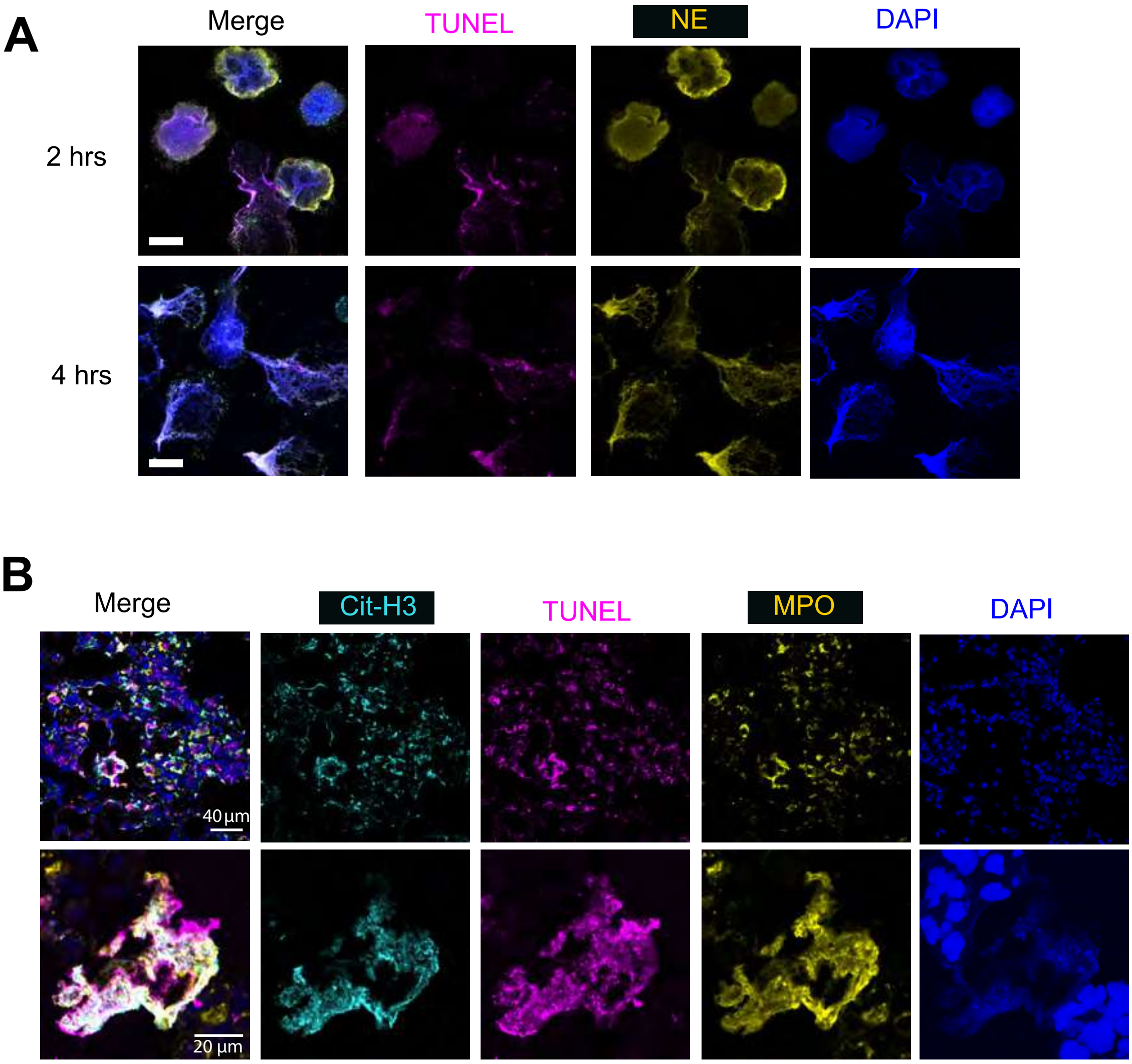

Fig. S2. Double stranded brakes occur during NETosis.
(**A**) Confocal fluorescence micrographs of human neutrophils activated with PMA for 2 hrs or 4 hrs, fixed and stained for TUNEL (magenta), NE (yellow) and DNA (DAPI, blue). Scale bars: 10 µm. (**B**) Confocal fluorescence micrographs of lung tissues from mice infected with *C. albicans*, 24 hrs post-infection, stained for citrullinated histone H3 (cit-H3, cyan), TUNEL (magenta), MPO (yellow) and DNA (DAPI, blue). Scale bars: 40 and 20 µm. Data are representative of 2 independent experiments.

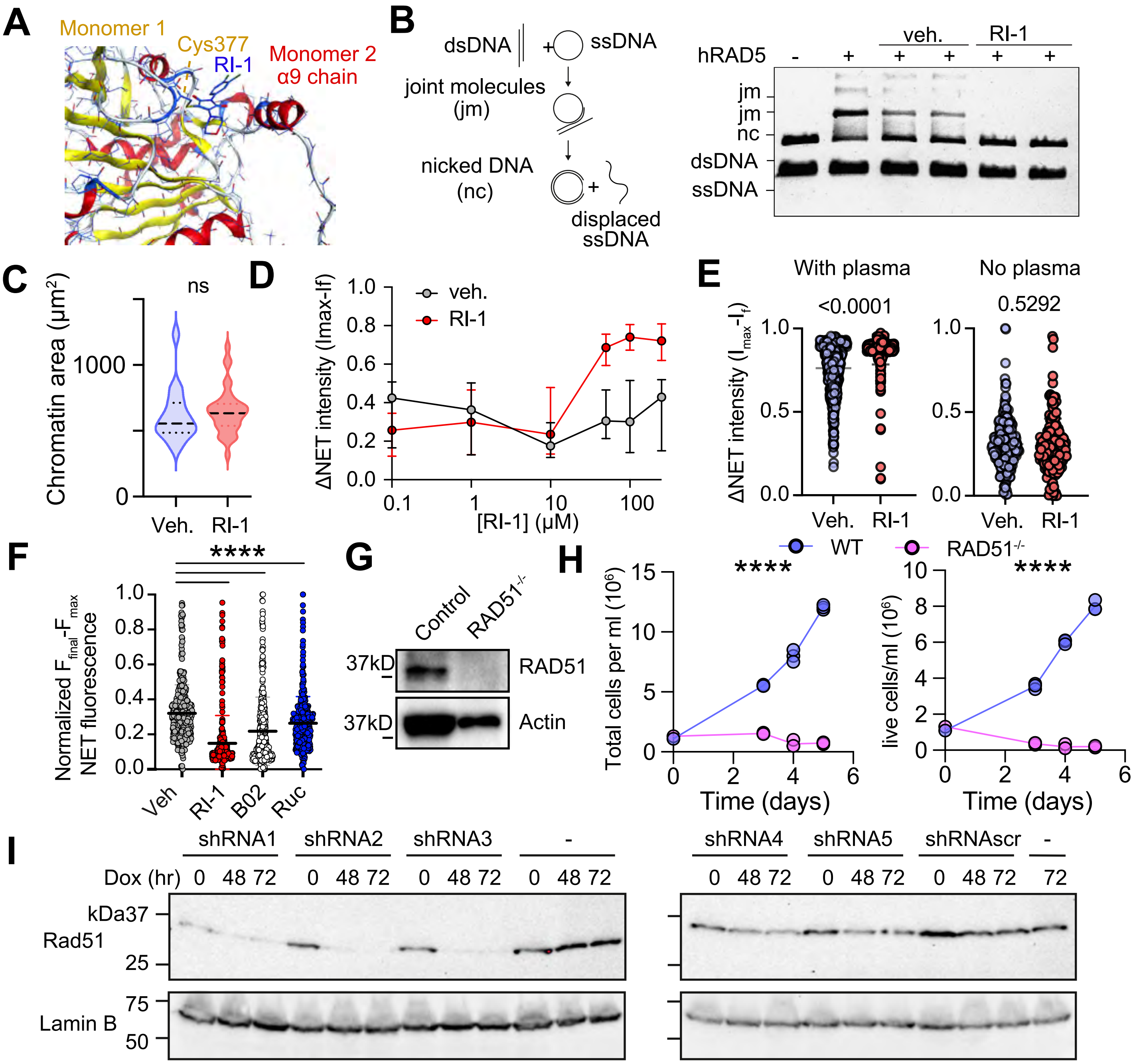

Fig. S3. Strategies to target RAD51 in neutrophils and HoxB8 hematopoietic progenitors.
(**A**) Illustration of RI-1 binding on a cysteine containing RAD51 protein-protein interface that is essential for the oligomerization of RAD51 monomers into filaments on single-stranded DNA. (**B**) DNA exchange assay showing that RI-1 inhibits RAD51 in vitro, as determined by agarose gel electrophoresis. (**C**) Quantification of extracellular chromatin decondensation in human neutrophils activated with PMA after 4 hrs in the presence of control vehicle or RI-1. (**D**) Changes in NET fluorescence intensity in the presence of control vehicle or different concentrations of RI-1. Data were acquired from replicate reactions involving hundreds of individual NET decay events over a 10 hr period. (**E**) Loss of NET fluorescence intensity measured by time-lapse microscopy over a 10-hr period in the absence or in the presence of 1% human plasma. Each point represents an individual NET. Data are representative of 2 independent experiments. (**F**) Loss in NET fluorescence intensity over 12 hrs of degradation in NETs formed by human blood neutrophils in the presence of, vehicle (DMSO), RI-1, B02 or Rucaparib. Data are representative of 3 independent experiments. (**G**) Western immunoblotting for RAD51 and actin proteins in whole cell extracts of either cultured HoxB8 immortalized hematopoietic progenitor WT control cells or cells where RAD51 was deleted by CRISPR. (**H**) Cell growth and viability over time of WT control cultured HoxB8 immortalized hematopoietic progenitor cells or cells where RAD51 was deleted by CRISPR. Data are representative of 3 independent experiments. (**I**) Western immunoblotting for RAD51 or Lamin B protein in cells infected with a viral vector carrying different inducible candidate silencing shRNAs against RAD51 or a scrambled sequence and induced with doxycycline for 0 hr, 48 hrs or 72 hrs. Statistics by one-way Anova (C-F) or two-way Anova (H): ****p<0.0001, ***p<0.001, **p<0.01, *p<0.05, ns>0.05.

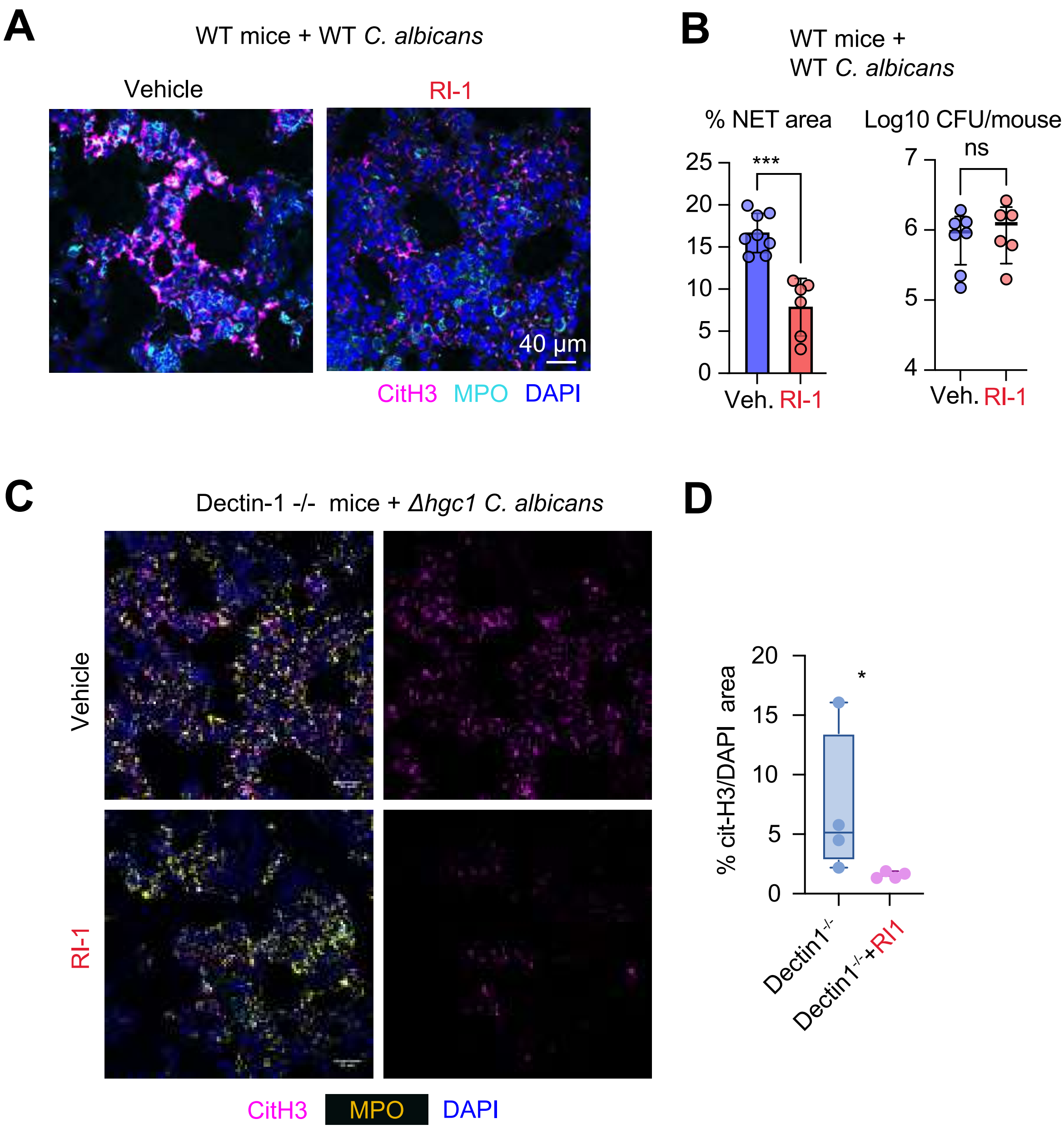

Fig. S4. RAD51 inhibition increases NET clearance in pulmonary *C. albicans* infection.
(**A**) Confocal fluorescence microscopy of lungs of WT mice treated with either vehicle or RI-1 and infected with WT *C. albicans*, 24 hrs post-infection, stained for citrullinated histone H3 (Cit-H3, magenta), and DNA (DAPI, blue). Scale bar: 40 µm. (**B**) Quantification of total NET area normalized to total lung tissue area (DAPI) and fungal load (CFU) in 8 control and 6 RI-1 treated mice. Each point represents an individual mouse. (**C**) Confocal fluorescence microscopy of lungs of *Dectin-1-/-* mice treated with either vehicle or RI-1 and infected with Δhgc1 C. albicans, 24 hrs post-infection, stained for citrullinated histone H3 (Cit-H3, magenta), and DNA (DAPI, blue). Scale bar: 30 µm. (**D**) Quantification of total NET area normalized to total lung tissue area (DAPI) in 4 control and 4 RI-1 treated*Dectin-1-/-* mice infected with*Δhgc1 C. albicans*, 24 hrs post-infection. Each point represents an individual mouse. (**A** to **D**) Data are representative of 2 independent experiments.
Statistics by T test: ****p<0.0001, ***p<0.001, **p<0.01, *p<0.05, ns>0.05.

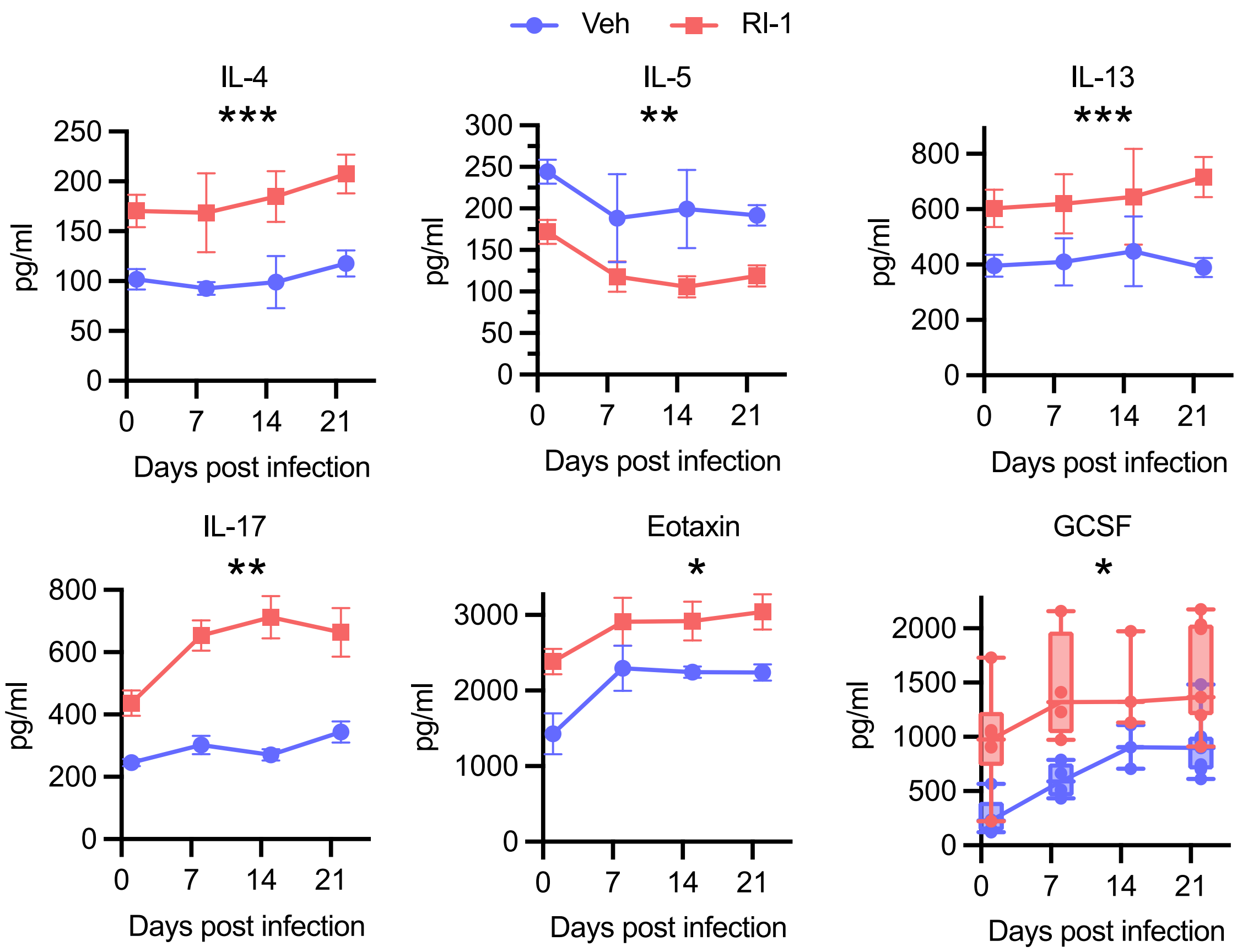

Fig. S5. Timeline of Cytokine induction in chronic murine aspergillosis.
Mean cytokine (IL-4, IL-5, IL-13, IL-17, GCSF) and chemokine (eotaxin) levels at 1, 8, 15, and 22 days post the initial infection in lung homogenates from mice receiving up to 4 weekly doses of intratracheal *A. fumigatus* and treated with either vehicle or RI-1. Individual values for each animal are shown for GCSF but summarized by mean and SD for all other variables. N=4 mice for all timepoints except at 15 days where lungs from N=3 mice where harvested. For IL-5, IL-4, IL-13, IL-17 and eotaxin each data point represents the mean with error bars depicting the ± SD. For G- CSF each point represents an individual animal with bars depicting the 10-90 percentile. Statistics of multiple grouped samples per timepoint by two-way Anova: ****$p<0.0001$, ***$p<0.001$, **$p<0.01$, *$p<0.05$, ns>0.05.

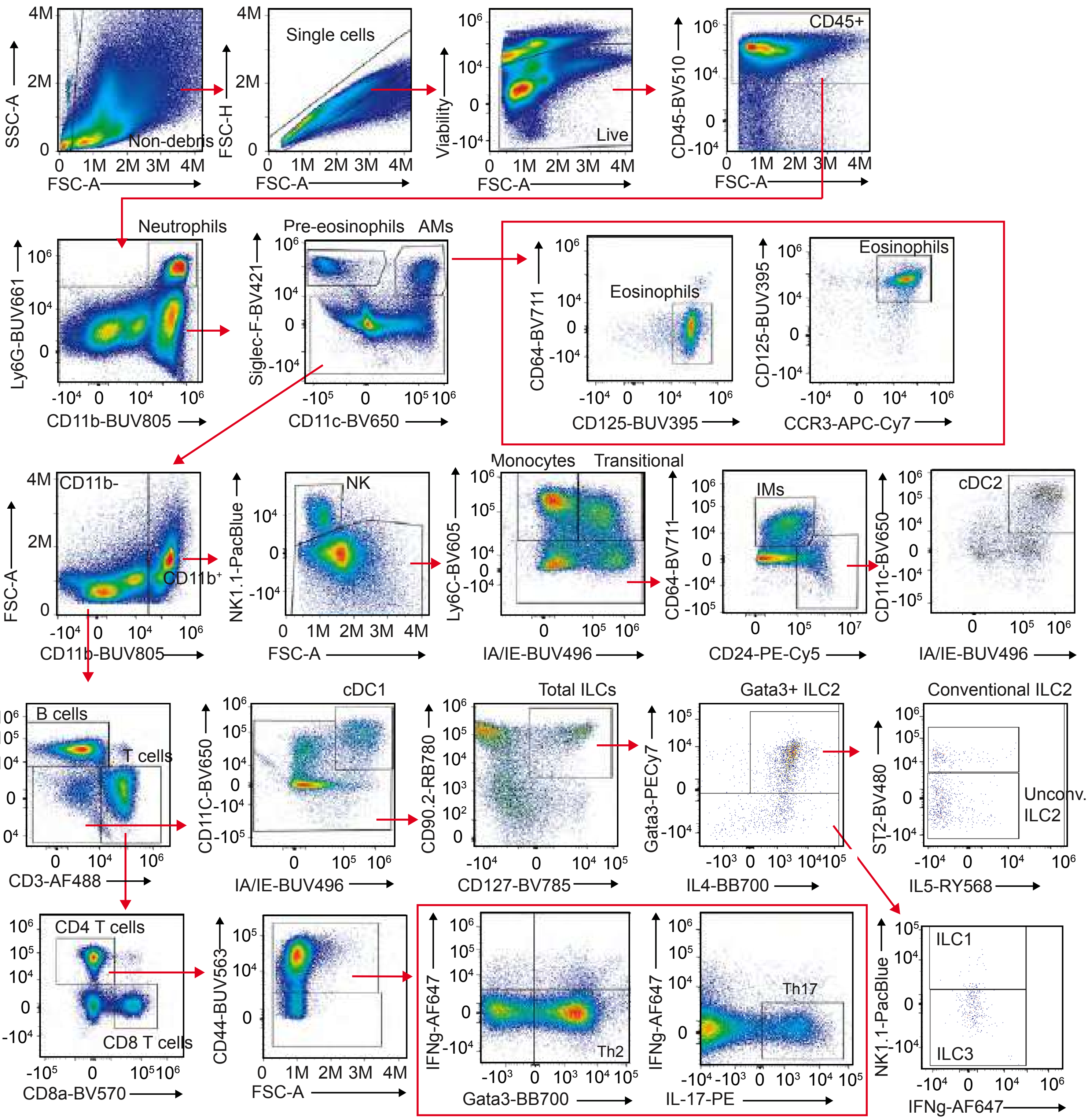

Fig. S6. Gating strategies for immune cell identification *in vivo*.
Gating strategies for the identification of neutrophils, eosinophils, monocytes, alveolar and interstitial macrophages, NK cells, dendritic cells, B cells, innate lymphocytes and T helper subsets from spectral flow cytometry data.

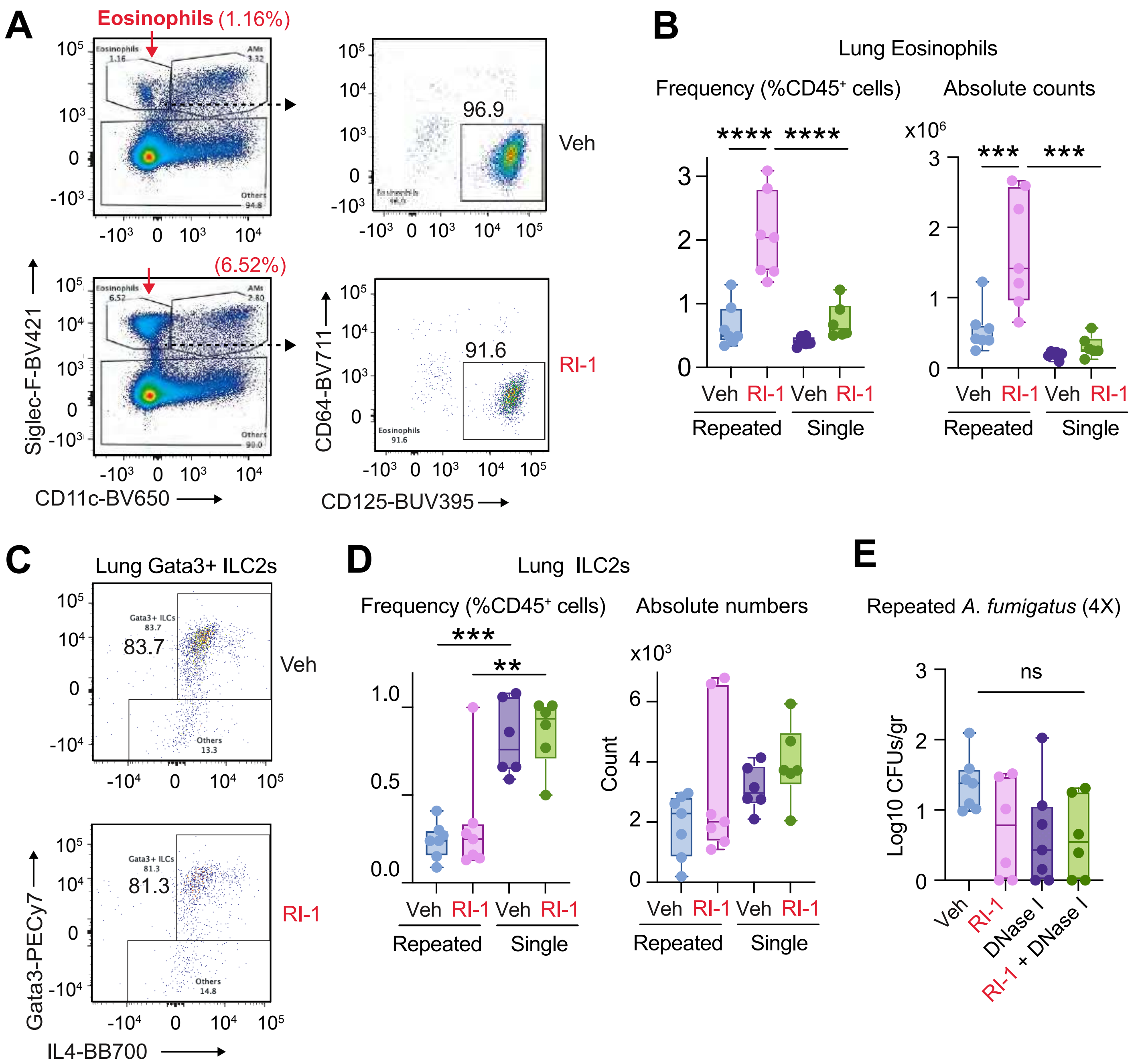

Fig. S7. Detailed analysis of lung eosinophils and IL2 cells.
(**A**) Representative flow cytometry plots depicting the gating strategy for eosinophil identification in WT mice treated with either vehicle or RI-1 and infected 4X with *A. fumigatus* 24 hrs post-infection. CD11c-, Siglec-Fhigh cells were subsequently gated for CD64 and CD125 to exclude CD64+ macrophages and select for CD64-,CD125+ eosinophil cells. Approximately 92-97% of the eosinophils in the first gate were confirmed not to be macrophages. Left panels also appear in (Fig. 3F). (**B**) Frequency and absolute counts of lung eosinophils in mice infected with 1 high dose or 4 lowerdoses of A. fumigatus, 24 hrs post infection. (**C**) Representative flow cytometry plots depicting GATA3+,IL-4+ eosinophils in the lungs of WT mice treated with either vehicle or RI-1 and infected 4X with A. fumigatus, 24 hrs post-infection. (**D**) Frequency and absolute counts of lung ILC2s in mice infected with 1 high dose (acute) or 4 lower doses (repeated) of A. fumigatus, 24 hrs post infection. (**E**) Fungal load in mice receiving or 4 lower doses (repeated) of A. fumigatus, 24 hrs post infection and treated with vehicle, or RI-1 alone or in combination with DNase I injection. Each data point represents an individual mouse in a single experiment, bars show the mean and 10%-90% percentile and error bars ± SEM. Data are representative of 3 independent experiments. Statistics by one-way Anova: ****p<0.0001, ***p<0.001, **p<0.01, *p<0.05, ns>0.05.

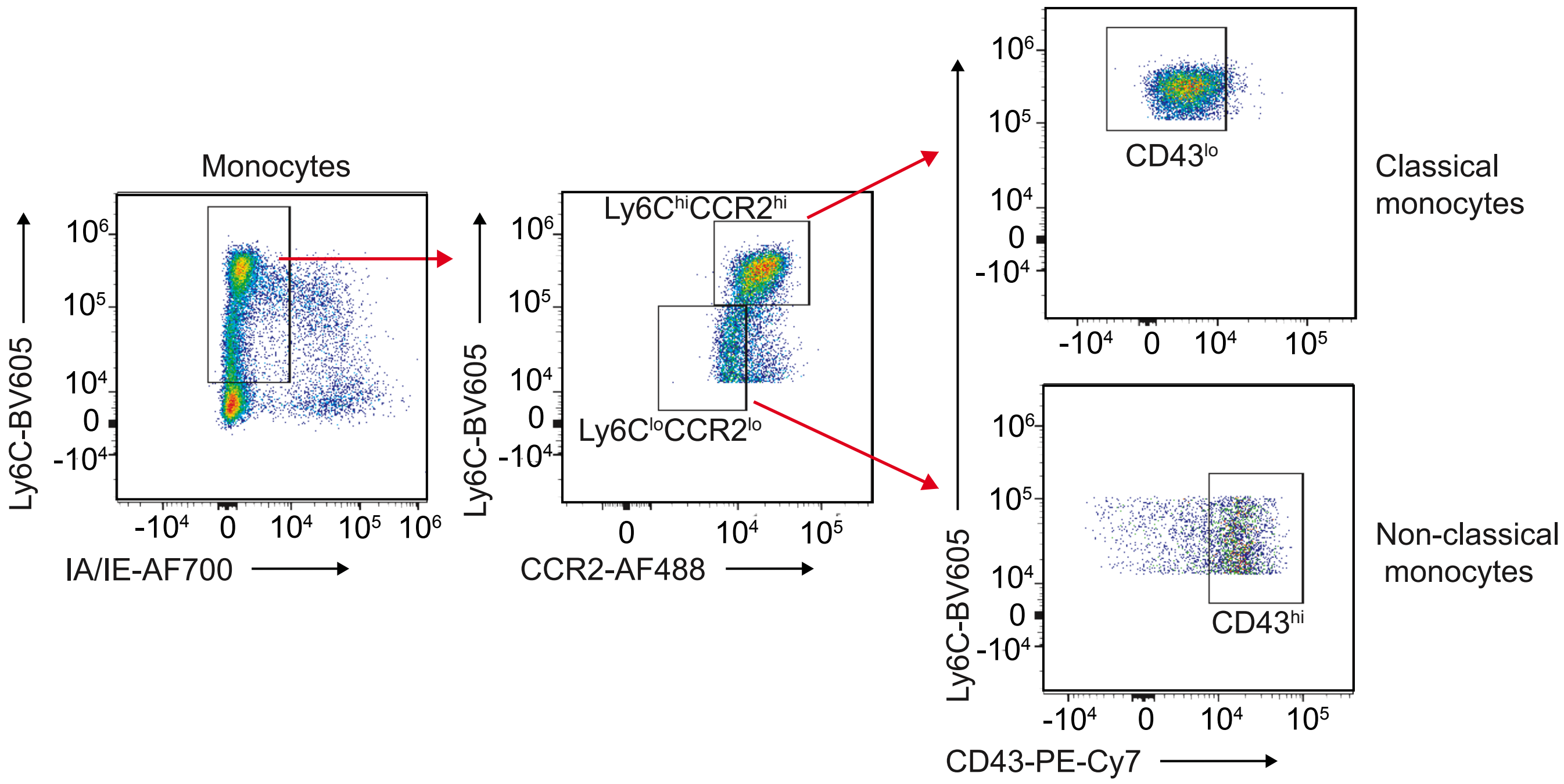

Fig. S8. Gating strategy for monocyte subset analysis.
Blood and lung monocytes were gated as classical inflammatory Ly6C^{high}, CCR2^{high}, CD43^{low} monocytes or non-classical Ly6C^{low}, CCR2^{low}, CD43^{low} monocytes.

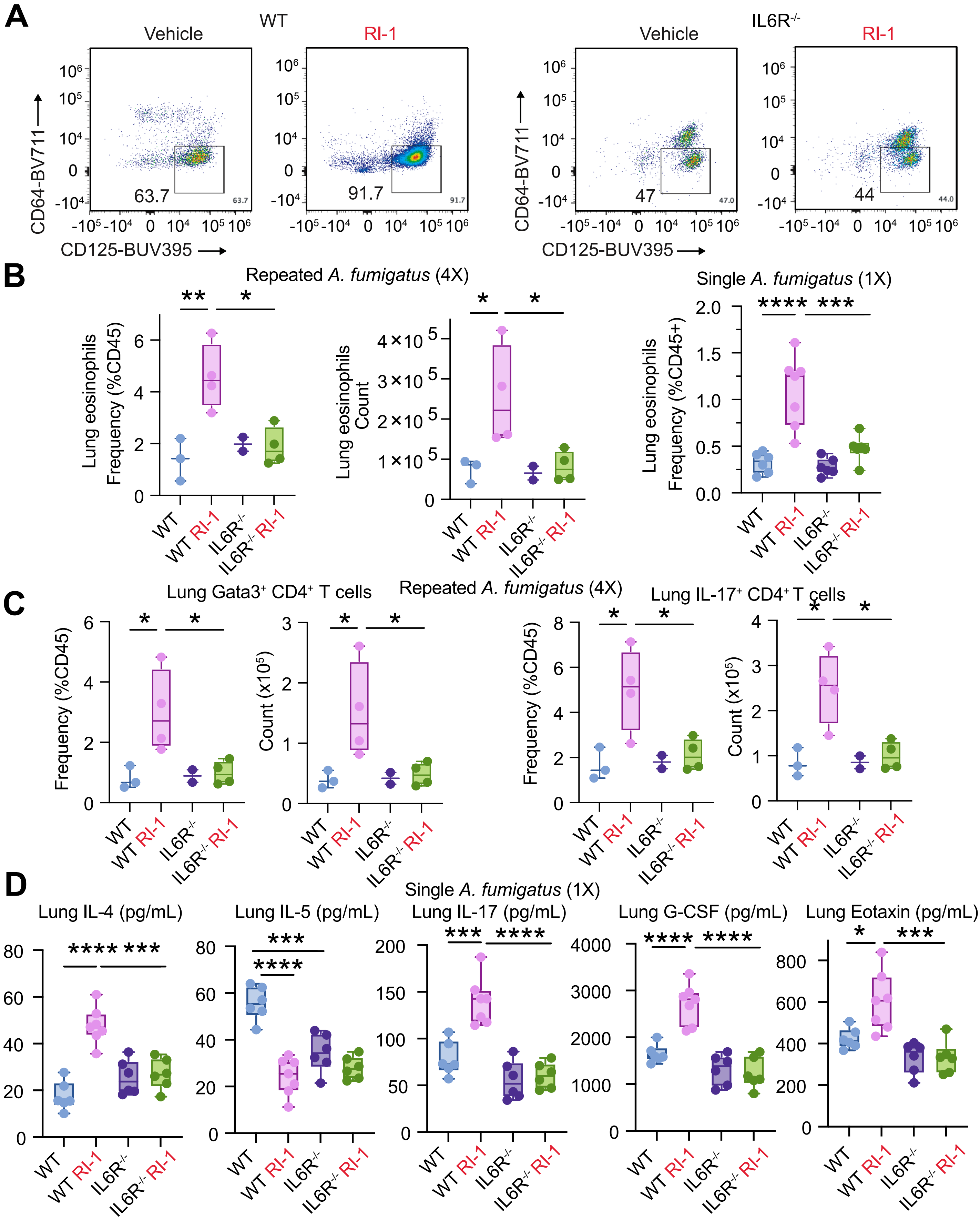

Fig. S9. Eosinophilia requires IL6 signalling.
(**A** to **D**) Analysis of lungs of WT and IL6R-/- mice treated with either vehicle or RI-1 and infected with either once (1X) or 4 weekly doses (4X) *A. fumigatus*, 24 hrs post-infection. (**A**) Flow cytometry plots depicting the separation of macrophages and eosinophils based on low CD64 and high CD125 surface marker expression of SiglecF^{high}, CD11c^{-} cells. (**B**) Frequency and absolute counts of lung eosinophils. (**C**) Frequency and absolute counts of lung Th2 and Th17 cells. (**D**) Cytokine concentrations in lung homogenates. Each data point represents an individual mouse in a single experiment. Bars show the mean and 10%-90% percentile and error bars ± SEM. Data are representative of 2 independent experiments. Statistics by one-way Anova: ****p<0.0001, ***p<0.001, **p<0.01, *p<0.05, ns>0.05.

Supplementary Table 1. List of reagents

Antibodies used for staining

CitH3	Abcam	ab5103
MPO	Bio-Techne	AF3667-goat
MPO	Abcam	ab208670-rabbit
MUC5AC	Abcam	AB3649
E-CADHERIN	BD Biosciences	610181
Neutrophil Elastase	GeneTex	GTX72042
donkey anti-goat 647	Invitrogen	A21447
donkey anti-rabbit 568	Invitrogen	A10042
donkey anti-mouse 647	Invitrogen	A31571
donkey anti-mouse 488	Invitrogen	A21202
Actin-phalloidin	Invitrogen	A12380
TUNEL (ThermoFisher, Click-iT™ TUNEL Alexa Fluor™ Imaging Assay)	Invitrogen	C10246
DAPI	Roche	10236276001
Hoechst	TargetMol	T5840
Sytox Green	Invitrogen	S7020
Sytox Orange	Invitrogen	S11368

Western Blot

RAD51	Abcam	ab213
ACTIN HRP	Cell signalling	5125S
MPO	Bio-Techne	AF3667
Lamin-B	Abcam	ab16048
Anti goat HRP	Invitrogen	PA1-28664
Anti mouse HRP	Invitrogen	31455
Anti rabbit HRP	Invitrogen	31463

Marker	Clone	Fluorophore	Company	Catalogue number
CCR2 (CD192)	Y15-488	AF488	BD Pharmingen	568414
CCR3 (CD193)	J073E5	APC/Cy7	Biolegend	144528
CD103	M290	PE-CF594	BD Horizon	565849
CD11b	M1/70	BUV805	BD Horizon	568345
CD11c	HL3	BV650	BD Horizon	564079
CD125	T21	BUV395	BD Optibuild	740324
CD127	SB/199	BUV737	BD Horizon	612841
CD127	SB/199	BV786	BD Pharmingen	563748
CD19	6D5	BV750	Biolegend	115561
CD24	M1/69	PE-Cy5	eBioscience	15-0242-82
CD3	17A2	AF488	Biolegend	100210
CD4	GK1.5	BV750	Biolegend	100467
CD4	RM4-5	BUV737	BD Horizon	612844
CD43	1B11	PE-Cy7	Biolegend	121218
CD44	IM7	BUV563	BD Horizon	569705
CD45	30-F11	BV510	Biolegend	103138
CD62L	MEL-14	PE/Dazzle 594	Biolegend	104448
CD64	X54-4/7.1	BV711	Biolegend	139311
CD64 (FcyRI)	X54-5/7.1	AF647	Biolegend	139322
CD69	H1.2F3	Spark PLUS B550	Biolegend	104562
CD8a	53-6.7	BV605	Biolegend	100744
CD8a	53-6.7	BV570	Biolegend	100740
CD90.2	53-2.1	RB780	BD Optibuild	755826
CTLA-4 (CD152)	UC10-4B9	PE/Fire810	Biolegend	106335
FOXP3	MF-14	AF700	Biolegend	126422
GATA3	L50-823	PE-Cy7	BD Pharmingen	560405
GATA3	L50-823	BB700	BD Horizon	566642
IFNg	XMG1.2	BV711	Biolegend	505836
IFNg	XMG1.2	AF647	BD Pharmingen	557735
IL-4	11B11	BV785	Biolegend	504141
IL-4	11B11	PE-Cy7	BD Pharmingen	560699
IL-5	TRFK5	RY586	BD Horizon	568530
IL-6	MP5-20F3	PE	BD Pharmingen	554401
Ly-6C	HK1.4	BV570	Biolegend	128030
Ly-6C	HK1.4	BV605	Biolegend	128036
Ly-6G	1A8	BUV661	BD Optibuild	741587
MHCII (I-A/I-E)	M5/114.15.2	BUV496	BD Optibuild	750281
NK1.1	PK136	Pacific Blue	Biolegend	108722
PD-1 (CD279)	29F.1A12	APC/Fire810	Biolegend	135252
Siglec-F	E50-2440	BV421	BD Horizon	562681
ST2	U29-93	BV480	BD Optibuild	746701

Other reagents

RI-1	MedChem Express	HY-15317
DNase I, bovine pancreas	Sigma Aldrich	11284932001
Rat anti-IL6R antibody	InVivoMab	BE0047
Rat IgG control	InVivoMab	BE0090
